# Application of diamondoids in source and maturity evaluation of light oil: a case study from the Kuqa Depression of the Tarim Basin, NW China

**DOI:** 10.1038/s41598-026-38619-z

**Published:** 2026-03-20

**Authors:** Haizu Zhang, Yiwen Sun, Yun Li, Yongfeng Zhu, Yongqiang Xiong, Ke Zhang, Baichuan Luo, Wenmin Jiang

**Affiliations:** 1 R＆D Center for Ultra-Deep Complex Reservoir Exploration and Development, CNPC, Korla, 841000 China; 2 Research Institute of Petroleum Exploration and Development, PetroChina, Tarim Oilfield Company, Korla, 841000 China; 3 State Key Laboratory of Deep Earth Processes and Resources, Guangzhou Institute of Geochemistry, Chinese Academy of Sciences, Guangzhou, 510640 China; 4 University of Chinese Academy of Sciences, Beijing, 100049 China

**Keywords:** Light oil, Diamondoids, Source facies, Maturity evaluation, Formation mechanism, Kuqa depression, Genetics, Solid Earth sciences

## Abstract

Determining the origin of light oil presents a significant challenge due to the diversity of genetic types and the limitation of conventional biomarkers. Diamondoid hydrocarbons, characterized by their thermal stability and enrichment in high maturity oil, are considered effective indicators for elucidating generation mechanisms and secondary alteration processes of light oils. In this study, the diamondoid distributions are examined in light oil samples from various regions (i.e., Wushi, Bozi, Dabei, Keshen, Kela, Dina, Dibei, Tuzi, Tudong, Yangtake, Yingmai, Hongqi, and Yaha) in the Kuqa Depression of the Tarim Basin, Northwest China, to discern the origin of the oils. Diamondoids concentrations and ratios in the Kuqa oils show significant variations, indicating two dominant source rock types and various maturity levels. Total diamondoid concentrations (including adamantanes, diamantanes, and triamantanes) vary from 141 to 19,137 ppm. Notably, the light oils from the Kela and Tuzi regions exhibit unusually high diamondoid concentrations (> 9000 ppm), while those from Yingmai, Hongqi, Yaha, Wushi, Keshen, and Tudong regions have relatively lower concentrations (< 2000 ppm). Employing our previously developed source facies discriminant model based on multivariate statistical analysis of multiple diamondoid indices, we suggest that the light oils from the Kela, Keshen, Yangtake, and Yaha regions are originated from lacustrine shales. In contrast, the other studied samples are inferred to primarily derive from coaly source rocks. Meanwhile, our maturity prediction model indicates that the maturity of the Kuqa oils ranges from 0.81 to 2.44 EASY%Ro. The highest maturity is found in the Kela light oils (2.3–2.5 EASY%Ro), while the lowest maturity is observed in the samples from the Wushi, Yingmai, and Hongqi areas (0.8–1.1 EASY%Ro). The formation mechanisms for some samples with anomalous diamondoid distributions are also explored. The Kela light oils, distinguished by their high maturity, abundant diamondoids, and relatively high biomarker concentrations, demonstrate a mixed origin with a notable contribution from highly mature condensates derived from the maturation of lacustrine kerogens. The Tuzi coal-derived oils, exhibiting moderate maturity (1.25% EASYRo) and characterized by elevated adamantane concentrations coupled with inconsistently relatively lower diamantane concentrations, are inferred to be condensates underwent evaporative (or migration) fractionation during upward migration from deeper reservoirs. Our findings corroborate the potential application of diamondoids as previously suggested. In summary, the diamondoid hydrocarbons provide a robust methodology for elucidating the origins, thermal maturity, and formation mechanisms of light oils, particularly within highly mature systems.

## Introduction

Light oil generally refers to crude oil with a density of 0.80 ~ 0.85 g/cm^3^ or American Petroleum Institute (API) gravity of 35 ~ 45°^1^. Condensate is the liquid hydrocarbons that condense from the oil and gas reservoirs due to the drop of pressure and temperature during petroleum exploitation. The condensate oil and gas reservoirs are a special type of reservoir that is between conventional oil and natural gas reservoirs, occurring in the state of gas phase under the subsurface reservoir conditions of high temperature and pressure. The density of condensate is generally less than 0.80 g/cm^3^ or more than 45° API, which also belongs to light oil literally. Light oils mostly are the thermal maturation products of kerogen and crude oil at high maturity stages (i.e., 1.3 ~ 2.0%Ro), which can also be considered as primary light oils. Hydrocarbon phase fractionation in the subsurface, caused by mixture, migration, evaporation, or gas washing, can also generate secondary condensates^2–7^. As the petroleum exploration progressively ventures into deep and ultra-deep strata, the light oil resource has emerged as a significant target in deep basins^8,9^. Given the advanced maturity of light oils, particularly condensates generated during primary expulsion, they often exhibit extremely low abundance even an absence of conventional biomarkers (i.e., terpanes and steranes). This makes it difficult to accurately identify and correlate biomarkers, significantly diminishing their role. Hence, new approaches and parameters need to be developed and introduced to investigate the source and maturity of light oil in deep reservoirs and highly mature petroleum systems.

Diamondoids—a group of three dimensionally fused cyclohexyl ring alkanes known for their diamond-like structures—demonstrate high stability and are enriched in highly mature oils, shales, and coals^10–13^. Numerous diamondoids-based indices have been constructed and applied in the petroleum geochemistry field. Among these, the diamondoid maturity indices, derived from the ratios of alkyl-substitutions on bridgehead to other positions, such as methyladamantane index (MAI) and methyldiamantane index (MDI) (see Table 2 for definitions), are paramount and recommended for the investigation of source rocks and oils at high maturity stages (i.e., Ro > 1.3%)^11,14–16^. The 4- + 3-methyldiamantanes (4- + 3-MDs) were initially considered to remain unaltered during natural oil cracking, and consequently utilized as a classic indicator for gauging the extent of crude oil cracking^12^. However, subsequent studies revealed that diamondoid hydrocarbons undergo thermal degradation at elevated maturity levels, thereby reducing their reliability beyond critical maturity thresholds^13,16–19^. Nevertheless, these compounds maintain diagnostic utility within the liquid hydrocarbon window. Moreover, diamondoids-based indices have been employed in establishing source-oil correlations^20–27^, discerning oil biodegradation^28–31^, tracing oil migration^32–34^, monitoring oil production^35^, identifying chemofacies^36^, fluid inclusion work^37^, and assessing evaporative fractionation^38–40^.

The Tarim Basin, northwest China, stands as an important basin for deep and ultra-deep petroleum exploration and exploitation. In recent years, significant discoveries of large-scale light oil accumulations have been made in the deep reservoirs of this basin. These light oil accumulations are mainly distributed in the Tabei Uplift, Tazhong Uplift, Southwest Depression, and Kuqa Depression^41–43^. The Kuqa Depression, accounting for over 90% of the proven natural gas reserves in the Tarim Basin, serves as an important gas-supplying base for China’s ‘West-East Gas Pipeline Project’^44,45^. It hosts multiple condensate gas fields and yields light oils alongside natural gas production. These hydrocarbons are predominantly believed to originate from the Triassic-Jurassic source rocks, which contain two main types of lacustrine mudstone and coal measure strata^41,42,46–48^. There are also ongoing controversies concerning the origin of oil and gas in the Kuqa Depression. For example, some researchers suggested that the condensates in the Kelasu structural belt were primarily originated from highly mature Triassic-Jurassic lacustrine shales^41,49,50^. Conversely, other researchers proposed that the condensates in this region were mainly derived from coal measures, with some contribution from lacustrine source rocks^48,51,52^. Therefore, the origin of light oils within the Kuqa Depression is notably more intricate than previously suggested. They may have multiple sources or mixed sources, and even could have undergone secondary alterations. As such, further researches for the identification and evaluation of the Kuqa Depression oil sources are still necessary.

In recent years, the diamondoids have also achieved significant applications in the light oils of the Tarim Basin, highlighting their importance^22,33,34,53–59^. In a study examining hydrocarbons for gas fields in the Kuqa Depression, Huang et al.^57^ found that diamondoid-based maturity ratios (i.e., MAI and MDI) have positive correlations with gas dryness (C_1_/ΣC_1−4_), concentrations of 4- + 3-MD, and concentrations of stable aromatics (i.e., naphthalene and phenanthrene) within a broad maturity range (i.e., Ro of 1.5–3.5%), suggesting that these ratios can serve as effective maturity parameters. The objective of this study is to ascertain the principal source and genetic mechanism of light oils in the Kuqa Depression of the Tarim Basin through systematical analysis of diamondoid indices employing our established source facies discriminant and maturity prediction models, combined with conventional biomarker data, thereby providing critical insights for future petroleum exploration.

## Geological settings

**Fig. 1:**
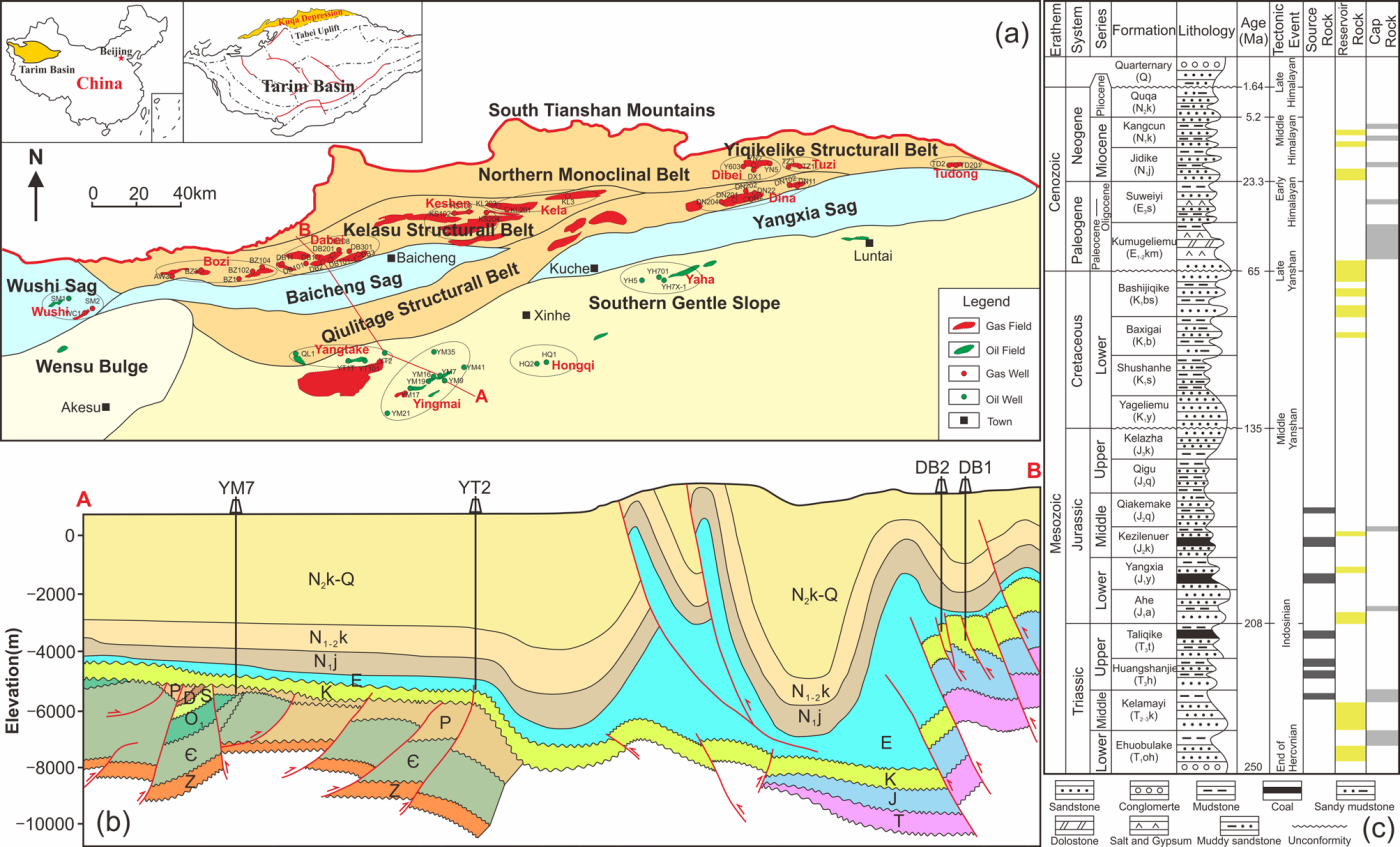
(**a**) Location and structural units of the Kuqa Depression showing sample locations; (**b**) structural cross-section of the Kuqa Depression along south-north trending, and (**c**) stratigraphic sequence of the Kuqa Depression (Modified after Guo et al.^60^ and Wang et al.^45^). The sample wells and region/field title within each black circle in Fig. 1a correspond to those listed in Table 1.

The Kuqa Depression in the northern Tarim Basin, NW China is located in the south of the Tianshan Mountains and north of the Tabei Uplift (Fig. 1). The depression is structurally NEE-trending with ~ 550 km from east to west and 30–80 km from north to south, covering an area of about 3.7 × 10^4^ km^2^. It can be divided into five structural belts (Northern monoclinal belt, Kelasu structure belt, Yiqikelike structural belt, Qiulitage structural belt, and Southern gentle slope) and three sags (Wushi, Baicheng, and Yangxia)^41,42,48^. The depression is a Meso-Cenozoic foreland basin developed on the base of the Paleozoic folded belt, and has experienced three evolution stages: a peripheral foreland basin stage during the Late Permian to the Middle Triassic, an extensional rift basin stage during the Late Triassic to the Middle Jurassic, and a rejuvenated foreland basin stage from the Cretaceous to Quaternary^41,46,48^.

During the extensional rift basin stage, over 1000 m of lacustrine and marginal lacustrine-swamp transitional sediments distributed across the Kuqa Depression, and the warm and humid climates facilitated the development of Triassic and Jurassic lacustrine and coal measure source rocks^41,46,48^. Since the Early Cretaceous, the depositional environments transformed to a shallow lacustrine with a relatively arid climate, and developed clastic sediments sourced from the north mountains in the rejuvenated foreland basin^46,48^. The intense compression and thrusting since the Miocene led to the development of a series of thrust belts from north to south and various fault-related folds, which finally evolved into a series of typical north-south oriented thrust structures following the southward uplift of the bottom slippage^46,48^.

The Kuqa Depression has deposited Meso-Cenozoic terrestrial stratigraphic sequence including Triassic, Jurassic, Cretaceous, Paleogene, Neogene, and Quaternary strata from bottom to top (Fig. 1c). The main source rocks were developed in the Middle Triassic to Middle Jurassic strata. These strata contain the lacustrine source rocks of the Middle-Upper Triassic Kelamayi Formation (T_2−3_k), the Upper Triassic Huangshanjie Formation (T_3_h), and the Middle Jurassic Qiakemake Formation (J_2_q). Coaly source rocks were found in the Upper Triassic Taliqike Formation (T_3_t), the Lower Jurassic Yangxia Formation (J_1_y), and the Middle Jurassic Kezilenuer Formation (J_2_k). Only the Middle Jurassic Qiakemake Formation (J_2_q) mainly contains lacustrine type II organic matter, whereas the remaining formations predominantly feature type III kerogen^41,46^. Deltaic sandstones, prevalent in Cretaceous to Cenozoic strata throughout the depression, served as potential reservoir rocks, with the Jurassic Ahe (J_1_a), Cretaceous Bashijiqike (K_1_bs), and Paleogene Kumugeliemu (E_1−2_km) formations being the primary reservoirs^45,48^. The salt and gypsum layer within the Paleogene Kumugeliemu Formation (E_1−2_km), a deposit formed as a result of marine transgression, functions as an effective regional seal and significantly impacts hydrocarbon accumulation^61^.

## Samples and methods

### Oil samples

In this study, 60 crude oil samples were collected at the well head after the separator and stored in sample vials. These samples were from different structural regions within the Kuqa Depression of the Tarim Basin: 4 samples from the Wushi Sag, 21 samples from the Kelasu structure belt, 8 samples from the Qiulitage structural belt, 9 samples from the Yiqikelike structural belt, and 18 samples from the Southern gentle slope (Fig. 1a). Most of the samples were produced from Jurassic (J), Cretaceous (K), and Paleogene (E) strata, while a small number were obtained from other strata, within the depths ranging from 1830 to 7331 m (Table 1). Most of the samples are light oil with density < 0.85 g/cm^3^ at 20℃ (Table 1), indicating relatively high maturity.

### Saturates, aromatics, resins, and asphaltenes (SARA) measurements

Approximately 30 mg oil sample was diluted in *n*-hexane, with α-cholestane-d_4_ injected as internal standard for quantification of terpanes and steranes. The asphaltene fraction in the oil sample was first precipitated by adding excess *n*-hexane. The resulting deasphaltened sample was subsequently separated into saturated, aromatic, and resin fractions via silica gel/alumina column chromatography employing sequential elution with *n*-hexane, *n*-hexane/dichloromethane mixture (3:2 v/v), and methanol. Following solvent evaporation at 50 ℃, the SARA fractions were quantified using a Sartorius CPA225D analytical balance with a measurement accuracy of ± 0.01 mg.

### Gas chromatography–mass spectrometry (GC–MS) analysis

The saturated fraction that containing internal standard was detected on an Agilent 7890 A/5977 GC–MS instrument, equipped with an HP-1 column (60 m × 0.32 mm i.d. × 0.25 μm film thickness). The GC oven temperature was maintained at 80 ℃ for 2 min, then elevated to 290 ℃ at 4 ℃/min, and finally sustained at 290 ℃ for 30 min. Helium was used as carrier gas at a constant flow rate of 1.2 mL/min. The mass spectrometer was operated in selected ion monitoring (SIM) mode. Relative response factors of individual compounds to the internal standard were all assumed to be one.

### Gas chromatography–triple quadrupole mass spectrometry (GC–MS/MS) analysis

A simple solvent dilution method was used to prepare sample for GC–MS/MS analysis to avoid the loss of diamondoids. Briefly, approximately 50 mg of each oil was spiked with three internal standards (i.e., *n*-dodecane-d_26_, *n*-hexadecane-d_34_, and α-cholestane-d_4_) and diluted in 4 mL isooctane within a glass vial. Following a 10 min ultrasonication and an additional 10 min centrifugation, the supernatant of the sample solution was transferred to a 2 mL glass vial for diamondoid analysis. GC–MS/MS analysis for diamondoids was conducted on a Thermo Fisher TSQ Quantum XLS instrument after the methods of Liang et al.^62^ and Xuan et al.^63^. An AS 3000 auto-sampler was used to inject sample into the GC instrument, which was equipped with a PTV injector and a DB-1 fused silica capillary column (50 m × 0.32 mm i.d. × 0.52 μm film thickness). Helium was used as a carrier gas at a constant flow rate of 1.5 mL/min. The GC oven temperature was held initially at 50 °C for 2 min, then ramped to 80 °C at 15 °C/min, 250 °C at 2.5 °C/min, and to 300 °C at 15 °C/min, and finally maintained at 300 °C for 10 min. The mass spectrometer was operated in selected reaction monitoring (SRM) mode. The quantification of diamondoid compounds was determined from the peak area ratio of the target compound to its corresponding internal standard, i.e., *n*-dodecane-d_26_ for adamantanes, and *n*-hexadecane-d_34_ for diamantanes, and α-cholestane-d_4_ for triamantanes. Calibration curves for individual diamondoids were established using a series of concentration-decreasing mixture solutions that contained three internal standards and thirteen pure diamondoid standards. Of these, eight adamantane standards (i.e., Adamantane, 1-Methyladamantane, 1,3-Dimethyladamantane, 1,3,5-Trimethyladamantane, 2-Methyladamantane, 1-Ethyladamantane, 1-Ethyl-3-Methyladamantane, and 2-Ethyladamantane) and two diamantane standards (i.e., Diamantane and 1-Methyldiamantane) were purchased from Chiron AS (Stiklestadveien, Norway)^62^, while three triamantane compounds (i.e., Triamantane, 9-Methyltriamantane, and 9,15-Dimethyltriamantane; purity of 97.5%) were prepared in-house by extraction from an artificially matured high-maturity crude oil using gas preparative chromatography^63^. A recent study^64^ proposed a pseudo-Multiple Reaction Monitoring (pMRM)–based GC–MS/MS approach for diamondoid detection, which demonstrated enhanced signal-to-noise ratio for higher diamondoids and improved sensitivity and selectivity relative to the classic MRM protocols^62,63^ under comparable sample preparation conditions. The newly developed diamondoid detection technique^64^ enhances molecular correlation capability in complex petroleum systems.

### Source facies and thermal maturity evaluation utilizing diamondoids

The source facies and thermal maturity of oil samples were determined through a source facies discriminant plot and maturity prediction function, which were established by multivariate statistical analysis on multiple diamondoid indices in our previous study^65^. Specifically, variations in abundance and distribution of diamondoids were obtained through thermal simulation experiments conducted on four types of organic matter (i.e., lacustrine Type I, II, and III kerogens, and non-biodegraded oil). Thermal maturity values of experimentally simulated samples were calculated using the EASY%Ro model developed by Sweeney and Burnham^66^, based on the documented time-temperature records from laboratory pyrolysis experiments. A total of 50 diamondoid indices of productions of thermal simulation experiments were calculated, comprising 32 relative abundance parameters of diamondoids, 9 isomerization ratios, and 9 concentration ratios (refer to Jiang et al.^65^ for details). Subsequently, principal component analysis (PCA) was applied to extract principal components from these standardized diamondoid indices. Discriminant analysis (DA) was performed on PCA results, which generated a source facies discriminant plot (Fig. 5) delineating four distinct fields corresponding to the four organic matter types in thermal simulation experiments. Concurrently, regression analysis (RA) was applied to the PCA results to establish a maturity prediction function correlating EASY%Ro values with diamondoid indices. The aforementioned 50 diamondoid indices were calculated and processed for oil samples from the Kuqa Depression following the methodology described by Jiang et al.^65^, thereby determining their source facies and thermal maturities.

## Results

### Biomarker parameters

The concentrations and parameters of biomarkers for light oils from the Kuqa Depression are presented in Table 1. The Kuqa Depression samples exhibit relatively low concentrations of C_29_ ααα 20R sterane (0–168ppm), with the most values below 20 ppm, indicating relatively high maturity levels. Relative abundances of regular steranes C_27_, C_28_, and C_29_ range from 5.4 to 37.8%, 11.6–37.9%, and 36.1–78.8%, respectively, with C_29_ regular steranes constituting the most abundant components, suggesting relatively high input of terrigenous higher plants.

### Diamondoid indices

**Fig. 2:**
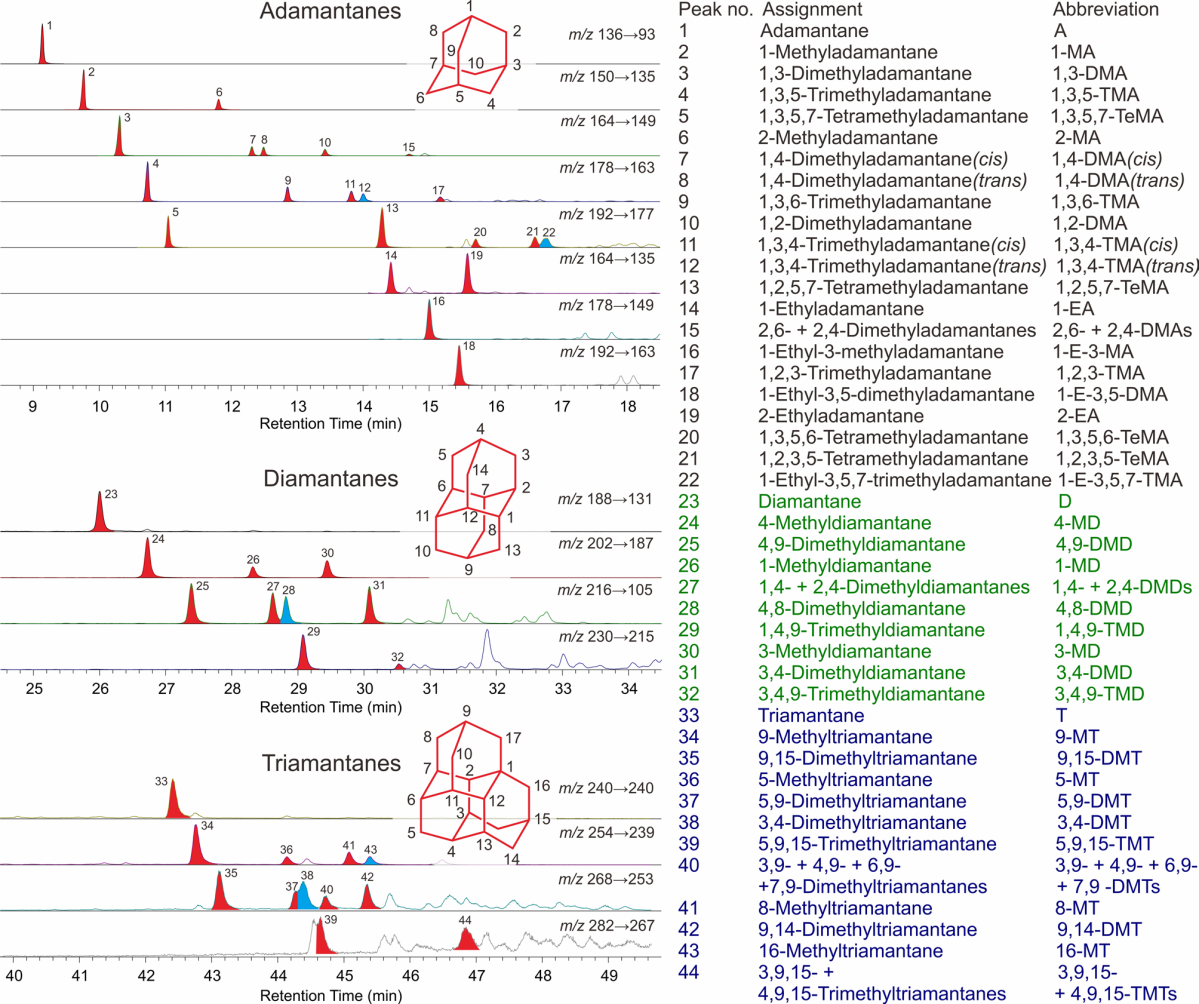
GC-MS/MS chromatograms showing adamantanes, diamantanes, and triamantanes in the sample KL203.

The diamondoid concentrations and indices for oils from the Kuqa Depression are presented in Table 2. Representative chromatograms of adamantanes, diamantanes, and triamantanes are illustrated in Fig. 2. The concentrations of adamantanes (As), diamantanes (Ds), triamantanes (Ts), and total diamondoids (including As, Ds, and Ts) were determined as 128–16363ppm, 11–2780ppm, 0–320ppm, and 141–19137ppm, respectively. The commonly used concentration parameter of 4- + 3-MDs for the Kuqa oils ranges from 5 to 1317ppm, in which most samples are lower than 200 ppm. Ten diamondoid isomerization ratios were calculated, with MAI, EAI, DMAI-1, DMAI-2, TMAI-1, TMAI-2, MDI, DMDI-1, DMDI-2, and MTI (see Table 2 for definitions) showing ranges of 0.39–0.82, 0.19–0.65, 0.28–0.84, 0.16–0.19, 0.21–0.62, 0.27–0.70, 0.11–0.58, 0.17–0.53, 0.29–0.66, and 0.28–0.61, respectively. The MAs/MDs concentration ratio varies between 1.3 and 14.5, with the minimum value observed in sample KL201-2 and the maximum value in sample TZ3.

### Source facies and thermal maturity values

Source facies and thermal maturity determined by the source facies discriminant plot and maturity prediction function are shown in Table 2; Figs. 5 and 7. The thermal maturity values of Kuqa oils range from 0.81 to 2.44 EASY%Ro. The Kela samples exhibit the highest maturity, followed by those from Dabei and Keshen. Samples from Bozi, Dina, Dibei, Tuzi, Tudong, Yangtake, and Yaha regions display comparable maturity levels. Notably, the Wushi, Yingmai, and Hongqi samples yield the lowest maturity values. The source facies discrimination results indicate that the Kela, Keshen, Yangtake, and Yaha samples are primarily derived from lacustrine type II kerogen, whereas the Wushi, Bozi, Dabei, Dina, Tuzi, Yingmai, and Hongqi samples are predominantly sourced from type III kerogen. The Dibei and Tudong samples demonstrate mixed contributions from both Type II and Type III kerogens.

**Table 1:** Basic data and specific biomarker parameters for oil samples from the Kuqa Depression.

Region/Field	Sample	Strata	Depth (m)	Saturates (%)	Aromatics (%)	Resins (%)	Asphaltenes (%)	Density (g/cm^3^)^1^	C_29_ ααα 20R Ste (ppm)^2^	C_27_ Ste (%)^3^	C_28_ Ste (%)^4^	C_29_ Ste (%)^5^	Source rock^6^
Wushi	SM1	K_1_s	5138.5–5143	71.11	12.89	8.44	7.11		33.0	31.4	26.2	42.5	Jurassic coal Measures^67^
	SM2	K_1_s	6002–6018	55.49	5.33	4.08	32.92		22.9	25.9	25.4	48.7	
	WC1-1	K	6002–6005	76.27	16.95	1.02	1.36		12.8	24.7	26.5	48.8	
	WC1-2	E	6038.5–6052	78.57	16.33	1.84	1.22		16.8	24.5	22.1	53.4	
Bozi	AW3	K	3518–3556	83.66	5.85	4.15	5.12		3.7	29.0	30.7	40.4	Jurassic coal Measures^42^
	BZ1	K_1_bs	7014–7084	85.31	6.94	4.49	2.04	0.791	4.0	24.0	29.4	46.6	
	BZ102	K_1_bs	6760–6885	80.51	9.64	0.21	1.03	0.801	1.3				
	BZ104	K_1_bs	6757–6850	85.48	5.37	0.83	0.48	0.794	3.7	32.6	26.5	40.9	
	BZ3	K_1_bs	5971.5–5985.5	84.16	7.12	0.31	0.27	0.808	1.8				
Dabei	DB101	E-K	5725–5783	85.37	5.11	2.38	3.40	0.795	1.9				Jurassic coal Measures^41,48^
	DB101-1	K_1_bs	5379–5384	83.48	5.43	1.30	8.70	0.797	4.8	24.6	31.0	44.4	
	DB1	K_1_bs	5460–5480	86.25	3.78	2.75	4.47	0.805	17.2	28.3	27.7	44.0	
	DB11	K_1_bs	5632–5716	85.52	6.44	0.59	0.59	0.804	4.2	23.5	29.3	47.2	
	DB2	K_1_bs	5577.5–5589	79.81	6.96	3.94	1.62	0.806	7.0	21.7	37.9	40.5	
	DB201	K_1_bs	5932–6145	85.95	4.25	3.27	3.60	0.813	7.8	24.6	28.2	47.2	
	DB208	K_1_bs	5755–5830	77.90	8.81	3.76	1.77	0.807	4.4	27.9	29.8	42.3	
	DB301	K_1_bs	6930–7012	75.00	7.57	7.89	6.25	0.806	6.4	28.5	21.8	49.7	
Keshen	KS102	K_1_bs	7210.5–7331.27	76.65	8.33	4.74	2.23	0.853	5.6	31.3	32.2	36.4	Triassic lacustrine Mudstones^50^
	KS103	E_2_k	6353.5–6366	82.89	5.92	2.41	0.83		20.4	20.0	33.3	46.7	
	KS204	K_1_bs	6810–6830	73.68	9.06	4.39	11.11	0.857	4.9	37.7	26.2	36.1	
Kela	KL201-1	E	3600–3607					0.849	12.8	25.1	29.8	45.1	Triassic lacustrine Mudstones^41,50,60^
	KL201-2	E	3630–3640					0.848	9.9	29.7	27.1	43.1	
	KL201-3	K_2_b	3665–3695	82.40	3.00	1.99	9.63	0.842	7.4	27.3	28.7	44.0	
	KL203	K_1_bs	3698.5–3716.5	95.70	2.84	1.46	0.00	0.839	41.8	27.4	28.3	44.3	
	KL3	E	3472–3479					0.840	108.2	21.3	35.4	43.3	
Dina	DN102	E	5745.5–5587.5	72.79	17.66	5.44	0.41	0.808	5.2	20.9	37.6	41.5	Jurassic coal measures^68^
	DN11	E	5328–5549	74.51	19.66	1.33	0.12	0.798	4.7	17.1	33.1	49.8	
	DN2	N_1_j	4597.44–4875.59	94.67	3.81	1.25	0.00		0.9	19.2	36.4	44.4	
	DN201	E_2−3_s	4780.50–4992.50	87.51	6.17	0.60	0.34	0.807	1.1	22.2	11.6	66.2	
	DN202	E_2−3_s	5022–5046	82.32	5.79	2.04	6.81	0.794	3.7	5.4	15.8	78.8	
	DN204	E_2−3_s	5001.5–5072					0.808	1.4	22.4	26.9	50.8	
	DN22	E_1−2_s	4748–4774	86.50	8.00	2.00	0.00	0.801	2.2	20.1	33.0	46.9	
Dibei	DX1	J_1_a	4708–4811.38	80.05	9.45	1.72	5.79	0.819	2.9	14.7	30.9	54.4	Triassic lacustrine and Jurassic coal mudstones^69,70^
	Y603	J_2_k_2_	471.2–489.2						42.9	17.6	26.9	55.5	
	YN2-1	J_1_y	4606–4620	82.00	7.33	4.33	3.33		18.2	19.2	27.8	53.0	
	YN2-2	J_1_a	4969–4982	54.18	2.68	1.68	37.46		5.7	17.9	31.1	51.0	
	YN5	J_1_y	4529.5–4538.5	70.27	8.45	4.72	13.51		168.0	20.6	34.3	45.1	
Tuzi	TZ1	E_1_k	2644–2644.21	80.33	4.92	1.97	9.84		0				Jurassic coal Measures^70^
	TZ3	N_1_p	1830.56–1832	89.08	7.34	1.31	0.00	0.807	1.3	32.3	24.1	43.6	
Tudong	TD2	J_1_y	3974–4000	77.24	17.57	0.57	0.25	0.810	4.2	15.1	30.3	54.6	Jurassic coal Measures^71^
	TD201	J_1_y	4150–4185	27.89	48.07	10.32	2.93	0.876	18.8	17.1	28.4	54.5	
Yangtake	QL1	E	5759.1–5969.87	71.20	7.95	3.97	14.90		19.3	26.3	26.8	46.9	Jurassic lacustrine Mudstones^47,72^
	YT1T	K	5309–5352	91.67	1.30	0.74	3.33	0.797	3.0	34.0	28.2	37.8	
	YT2	K	5327.5–5401.75	86.32	1.89	1.18	8.49		17.8	29.4	25.2	45.5	
	YT101	K	5350.5–5355.5	72.61	2.31	1.65	20.47		10.2	31.4	25.6	43.0	
Yingmai	YM7	E	4690–4700	87.94	3.22	4.56	3.23	0.777	16.1	37.8	25.2	37.0	Triassic lacustrine and Jurassic coal mudstones^41,47,73^
	YM9	E	4683–4690	86.44	3.52	3.77	4.27	0.760	4.3	35.8	23.2	41.0	
	YM16	E	4683–4692	82.00	4.50	3.25	8.00	0.799	10.1	36.6	23.2	40.3	
	YM17	E	4635.03–4671.24	76.25	12.36	2.89	3.86	0.783	34.8	32.6	23.9	43.6	
	YM19	E	4663.75–4678.66					0.776	32.7	31.9	23.7	44.4	
	YM21	E	4452.5–4461.5	83.22	2.31	3.95	7.24	0.775	3.3	35.4	24.0	40.6	
	YM35	S	5623–5626	62.42	11.07	6.71	19.46	0.831	68.4	30.3	24.0	45.7	
	YM41	S	5287.95	58.91	16.34	8.42	15.84	0.835	35.2	30.6	23.9	45.6	
Hongqi	HQ1	E	4572.5–4575.5	73.90	8.21	5.28	8.21		9.3	28.3	29.7	41.9	lacustrine and coal mudstones^74^
	HQ2	E	4558–4559	68.49	6.16	4.79	17.47		12.6	28.3	28.3	43.4	
Yaha	YH5-1	N_1_j	5031.23–5103	91.18	3.38	2.63	0	0.813	30.2	30.5	23.9	45.7	Triassic lacustrine Mudstones^41,47^
	YH5-2	O	5810.5–5807	74.31	12.15	3.62	0.96	0.822	15.5	29.4	26.8	43.8	
	YH701-1	E	5160–5190	79.28	8.29	2.21	7.73	0.818	29.4	29.7	23.1	47.2	
	YH701-2	E	5203–5206	63.43	16.67	7.21	6.22	0.823	29.6	29.7	24.7	45.6	
	YH7x-1	Є-O	5818.5–5826.4	70.65	14.30	3.76	1.29	0.843	2.7	32.7	30.3	37.0	

1: density in g/cm^3^ at 20 °C; 2: concentration of C_29_ ααα 20R sterane (ppm). The typical quantification limit for steranes determined by SIM–GC–MS was 1.5 ppm, suggesting that sterane concentrations approaching the 3.0 ppm threshold may be subject to potential analytical noise. 3: C_27_ Steranes (%) = C_27_ Regular steranes/(C_27_ + C_28_ + C_29_) Regular steranes; 4: C_28_ Steranes (%) = C_28_ Regular steranes/(C_27_ + C_28_ + C_29_) Regular steranes; 5: C_29_ Steranes (%) = C_29_ Regular steranes/(C_27_ + C_28_ + C_29_) Regular steranes; 6: source rock for the studied area identified by conventional biomarkers in previous work.

**Table 2:** Diamondoid indices for oils samples from the Kuqa Depression.

Sample	1	2	3	4	5	6	7	8	9	10	11	12	13	14	15	16	17	18	19
SM1	465	26	1.1	492	14.0	0.46	0.23	0.49	0.30	0.22	0.31	0.28	0.20	0.53	0.41	6.3	1.13	1.13	III
SM2	681	29	1.0	711	15.9	0.45	0.23	0.49	0.28	0.22	0.31	0.31	0.24	0.60	0.38	8.4	1.19	0.86	III
WC1-1	697	30	1.0	728	16.1	0.44	0.22	0.46	0.27	0.23	0.31	0.31	0.23	0.57	0.43	8.0	1.19	0.86	III
WC1-2	727	34	0.8	762	18.4	0.43	0.22	0.45	0.26	0.21	0.29	0.29	0.19	0.46	0.41	7.4	1.14	0.87	III
AW3	3590	187	11.7	3789	95.8	0.57	0.35	0.63	0.41	0.38	0.43	0.34	0.27	0.58	0.41	8.6	1.28	1.53	III
BZ1	3250	156	9.1	3415	79.9	0.54	0.35	0.58	0.36	0.31	0.38	0.32	0.24	0.54	0.37	8.4	1.22	1.26	III
BZ102	2563	128	8.4	2700	65.7	0.52	0.33	0.54	0.34	0.27	0.35	0.31	0.23	0.55	0.39	7.2	1.19	1.19	III
BZ104	3780	213	12.0	4004	108.8	0.56	0.34	0.59	0.38	0.31	0.39	0.37	0.26	0.55	0.44	6.5	1.35	1.39	III
BZ3	1468	57	0.1	1526	29.6	0.48	0.27	0.51	0.31	0.25	0.32	0.33	0.24	0.53	/	8.7	1.25	0.81	III
DB101	5349	289	18.8	5658	145.2	0.70	0.42	0.84	0.65	0.60	0.62	0.41	0.36	0.63	0.46	7.9	1.44	2.01	III
DB101-1	4983	316	19.8	5319	159.3	0.70	0.42	0.77	0.53	0.53	0.53	0.42	0.38	0.65	0.44	7.5	1.45	1.96	III
DB1	5003	335	21.4	5360	168.2	0.67	0.40	0.77	0.49	0.52	0.51	0.42	0.38	0.64	0.51	7.0	1.46	1.95	III
DB11	4797	301	18.7	5117	149.5	0.68	0.41	0.79	0.53	0.54	0.54	0.43	0.37	0.65	0.47	7.5	1.49	1.96	III
DB2	4718	322	21.5	5062	158.9	0.68	0.42	0.78	0.51	0.52	0.52	0.41	0.37	0.63	0.46	6.8	1.43	1.97	III
DB201	4813	453	29.9	5296	222.5	0.67	0.41	0.77	0.52	0.50	0.53	0.42	0.37	0.63	0.49	4.8	1.45	2.06	III
DB208	4516	332	24.8	4872	165.8	0.71	0.50	0.81	0.56	0.56	0.58	0.44	0.41	0.66	0.47	6.2	1.51	2.08	III
DB301	13,345	1080	69.3	14,494	535.6	0.71	0.52	0.80	0.53	0.53	0.54	0.42	0.37	0.63	0.47	5.6	1.47	2.07	III
KS102	856	141	14.6	1011	71.6	0.65	0.37	0.69	0.55	0.43	0.58	0.31	0.46	0.56	0.55	1.7	1.20	2.09	I
KS103	128	11	0.8	141	5.1	0.51	0.27	0.28	0.16	0.25	0.37	0.37	0.37	0.56	0.57	4.8	1.34	1.50	II
KS204	710	118	12.4	841	61.2	0.59	0.35	0.61	0.46	0.35	0.50	0.21	0.40	0.48	0.51	1.8	0.94	1.96	I
KL201-1	10,203	1925	214.4	12,343	910.4	0.79	0.62	0.83	0.68	0.59	0.68	0.58	0.52	0.58	0.58	2.0	1.84	2.40	II
KL201-2	10,305	2780	319.5	13,404	1317.1	0.77	0.61	0.82	0.67	0.56	0.67	0.57	0.51	0.58	0.59	1.3	1.84	2.44	I
KL201-3	15,540	2602	297.0	18,439	1238.9	0.80	0.63	0.83	0.68	0.61	0.70	0.58	0.52	0.60	0.59	2.5	1.85	2.40	II
KL203	16,363	2496	278.4	19,137	1187.8	0.82	0.65	0.84	0.69	0.62	0.70	0.58	0.53	0.61	0.60	2.8	1.85	2.39	II
KL3	937	193	11.2	1141	86.3	0.73	0.40	0.79	0.63	0.54	0.66	0.53	0.47	0.59	0.61	1.8	1.72	2.33	II
DN102	4778	211	5.7	4995	109.8	0.56	0.34	0.67	0.38	0.36	0.35	0.30	0.25	0.60	0.28	9.6	1.18	1.33	III
DN11	4164	193	6.1	4363	102.0	0.55	0.34	0.70	0.39	0.38	0.36	0.33	0.27	0.64	0.34	9.2	1.23	1.42	III
DN2	5251	304	9.4	5565	161.4	0.52	0.32	0.66	0.36	0.35	0.33	0.30	0.24	0.64	0.30	6.5	1.16	1.46	III
DN201	4224	172	4.1	4400	91.4	0.54	0.32	0.68	0.36	0.35	0.34	0.30	0.24	0.61	0.30	10.3	1.16	1.21	III
DN202	5448	241	7.2	5696	128.5	0.54	0.30	0.68	0.37	0.38	0.35	0.30	0.23	0.58	0.31	9.4	1.16	1.30	III
DN204	4875	200	5.8	5081	105.4	0.54	0.31	0.69	0.38	0.38	0.35	0.30	0.24	0.60	0.33	10.5	1.18	1.27	III
DN22	3661	153	4.6	3819	81.5	0.45	0.29	0.50	0.31	0.25	0.29	0.30	0.21	0.57	0.33	7.6	1.18	0.87	III
DX1	2168	107	3.7	2279	53.7	0.53	0.31	0.55	0.35	0.29	0.37	0.33	0.26	0.55	0.35	8.0	1.25	1.25	III
Y603	3677	212	13.1	3903	104.3	0.57	0.31	0.68	0.45	0.36	0.44	0.41	0.39	0.63	0.46	5.9	1.43	1.58	III
YN2-1	545	26	0.5	572	12.3	0.39	0.19	0.43	0.25	0.21	0.27	0.25	0.28	0.56	/	6.5	1.05	0.81	II
YN2-2	1630	97	3.5	1731	50.6	0.52	0.28	0.54	0.34	0.28	0.36	0.33	0.25	0.53	0.34	6.2	1.24	1.34	III
YN5	462	25	0.5	487	11.0	0.43	0.21	0.46	0.27	0.22	0.29	0.17	0.27	0.45	/	6.2	0.86	0.94	II
TZ1	9365	463	23.1	9852	239.2	0.56	0.45	0.58	0.38	0.27	0.32	0.32	0.25	0.56	0.31	6.5	1.22	1.25	III
TZ3	14,046	333	5.0	14,384	169.7	0.59	0.45	0.58	0.37	0.32	0.36	0.39	0.30	0.58	0.40	14.5	1.39	1.25	III
TD2	981	49	1.9	1032	25.7	0.47	0.26	0.51	0.31	0.23	0.31	0.29	0.26	0.56	0.37	6.6	1.14	1.06	III
TD201	707	50	2.5	760	25.1	0.48	0.27	0.52	0.33	0.24	0.32	0.30	0.28	0.58	0.39	4.8	1.18	1.36	II
QL1	732	53	4.2	789	28.8	0.48	0.27	0.53	0.33	0.24	0.34	0.31	0.24	0.56	0.38	4.9	1.19	1.40	II
YT1T	3049	177	9.4	3235	93.0	0.53	0.33	0.54	0.33	0.27	0.35	0.33	0.24	0.55	0.38	5.8	1.25	1.29	II
YT2	3549	204	10.9	3764	105.5	0.53	0.34	0.52	0.32	0.28	0.35	0.34	0.24	0.56	0.39	5.7	1.26	1.26	II
YT101	2461	142	8.4	2612	75.2	0.53	0.32	0.54	0.33	0.27	0.36	0.33	0.23	0.54	0.39	5.9	1.24	1.29	II
YM7	1411	62	2.9	1476	31.6	0.57	0.33	0.56	0.34	0.28	0.36	0.35	0.26	0.51	0.38	7.9	1.29	1.11	III
YM9	1751	80	3.3	1834	42.2	0.58	0.34	0.56	0.34	0.29	0.37	0.35	0.23	0.50	0.39	7.5	1.30	1.15	III
YM16	1685	64	2.5	1752	33.3	0.58	0.32	0.56	0.34	0.30	0.38	0.37	0.25	0.53	0.41	9.0	1.34	1.01	III
YM17	1387	60	2.7	1450	31.6	0.56	0.33	0.56	0.34	0.29	0.37	0.36	0.24	0.52	0.42	7.6	1.32	1.10	III
YM19	1519	62	3.1	1585	31.9	0.59	0.33	0.58	0.36	0.30	0.38	0.36	0.25	0.52	0.40	8.5	1.31	1.09	III
YM21	1851	75	2.9	1929	38.9	0.56	0.33	0.53	0.32	0.30	0.37	0.36	0.24	0.54	0.41	8.8	1.30	1.03	III
YM35	237	11	0.0	248	5.6	0.44	0.21	0.51	0.30	0.23	0.32	0.30	0.17	0.40	/	8.0	1.16	1.01	III
YM41	451	23	0.9	475	11.7	0.47	0.23	0.51	0.30	0.24	0.33	0.31	0.22	0.56	0.50	7.5	1.19	1.06	III
HQ1	1601	64	2.2	1667	34.5	0.51	0.27	0.50	0.30	0.25	0.33	0.34	0.21	0.54	0.36	9.3	1.26	0.87	III
HQ2	1515	62	2.2	1579	32.6	0.51	0.27	0.50	0.30	0.25	0.34	0.33	0.22	0.49	0.35	8.9	1.25	0.91	III
YH5-1	1448	95	5.5	1549	50.5	0.50	0.27	0.51	0.31	0.25	0.33	0.39	0.25	0.54	0.43	5.2	1.38	1.31	II
YH5-2	396	29	1.8	427	14.1	0.46	0.23	0.50	0.31	0.23	0.33	0.23	0.22	0.41	0.51	5.3	0.99	1.31	II
YH701-1	1439	88	5.4	1533	46.3	0.50	0.26	0.52	0.31	0.24	0.31	0.35	0.25	0.55	0.40	5.5	1.29	1.24	II
YH701-2	1402	100	6.9	1509	52.4	0.50	0.26	0.52	0.31	0.24	0.32	0.36	0.25	0.53	0.43	4.6	1.31	1.35	II
YH7x-1	384	35	1.1	420	14.2	0.43	0.24	0.45	0.28	0.21	0.30	0.11	0.20	0.29	0.54	4.4	0.71	1.34	II

1: concentration of total adamantanes (ppm); 2: concentration of total diamantanes (ppm); 3: concentration of total triamantanes (ppm); 4: concentration of total diamondoids (ppm), including adamantanes, diamantanes, and triamantanes; 5: concentration of 4- +3-methyldiamantanes (4- +3-MDs, ppm); 6: MAI = 1-MA/(1-MA + 2-MA); 7: EAI = 1-EA/(1-EA + 2-EA) ; 8: DMAI-1 = 1,3-DMA/(1,3-DMA + 1,2-DMA); 9: DMAI-2 = 1,3-DMA/(1,3-DMA + 1,4-DMA); 10: TMAI-1 = 1,3,5-TMA/(1,3,5-TMA + 1,3,4-TMA); 11: TMAI-2 = 1,3,5-TMA/(1,3,5-TMA + 1,3,6-TMA); 12: MDI = 4-MD/(4-MD + 1-MD + 3-MD); 13: DMDI-1 = 4,9-DMD/(4,9-DMD + 3,4-DMD); 14: DMDI-2 = 4,9-DMD/(4,9-DMD + 4,8-DMD); 15: MTI = 9-MT/(9-MT + 5-MT + 8-MT + 16-MT); 16: MAs/MDs = (1-MA + 2-MA)/(4-MD + 1-MD + 3-MD); 17: %Rc = 2.4389 × MDI + 0.4363, proposed by Chen et al.^11^; 18: EASY%Ro, calculated based on the work of Jiang et al.^65^; 19: source facies, determined based on the work of Jiang et al.^65^.

## Discussion

### Diamondoid concentrations and groups of the Kuqa light oils

**Fig. 3:**
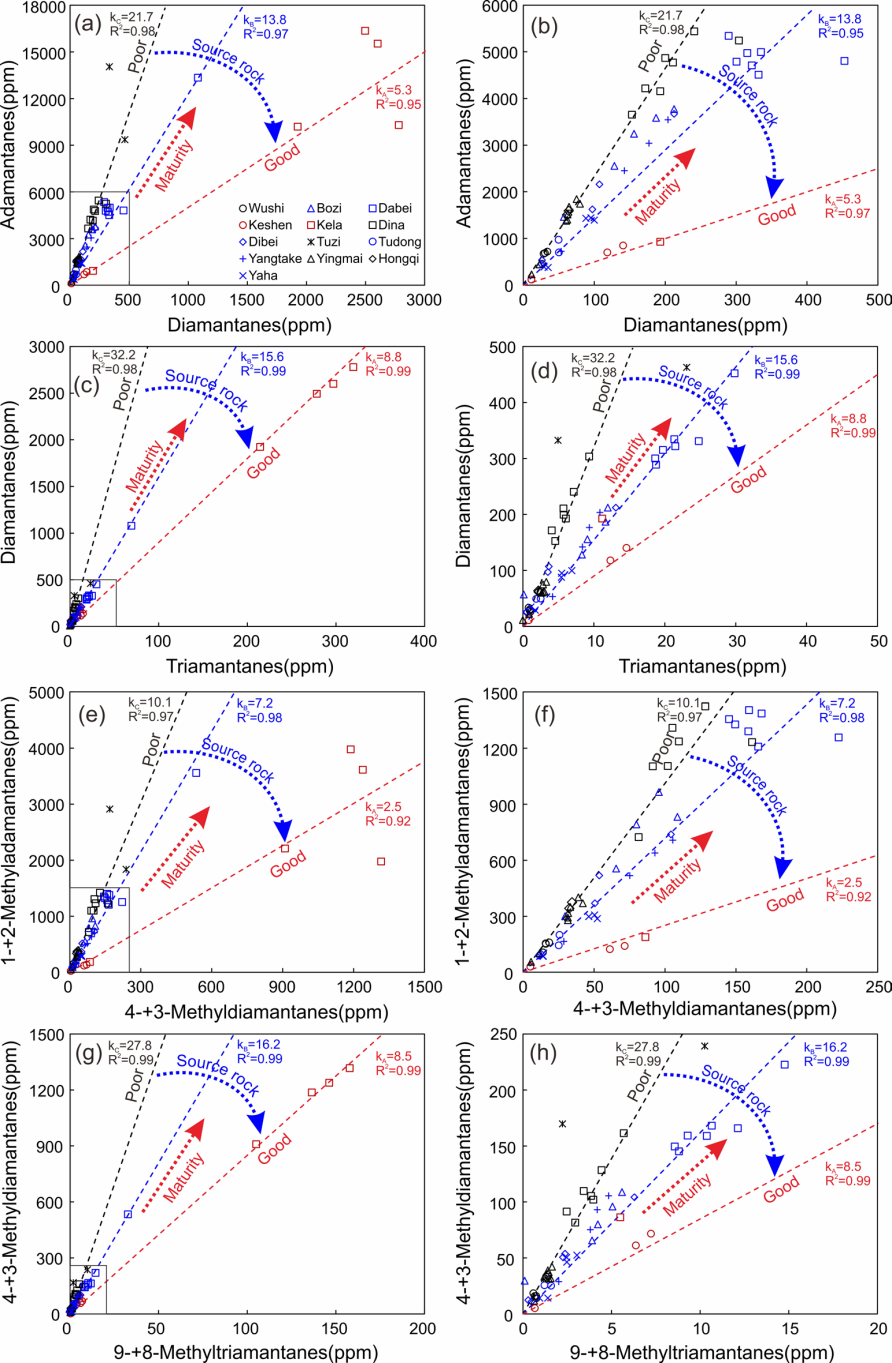
Crossplots of concentrations of adamantanes vs. diamantanes (**a**,**b**), diamantanes vs. triamantanes (**c**,**d**), 1- + 2-Methyladamantanes vs. 4- + 3-Methyldiamantanes (**e**,**f**), and 4- + 3-Methyldiamantanes vs. 9- + 8-Methyltriamantanes (**g**,**h**) for light oils in the Kuqa Depression. The different colored regression lines correspond to sample groups plotted in the same colors. Trend line and R^2^ value of linear fit for samples from Keshen and Kela (Group A; red samples) are depicted in red, that for samples from Bozi, Dabei, Dibei, Tudong, Yangtake, and Yaha (Group B; blue samples) are illustrated in blue, and that for samples from Wushi, Dina, Yingmai, and Hongqi (Group C; black samples) are represented in black. Notably, the Tuzi samples do not align with any of these delineated categories as they are far away from any of these fitting lines.

As illustrated in Fig. 3, the light oils from the Kuqa Depression exhibit significant variations in diamondoid concentrations. Notably, the samples from Kela field (except for KL3) exhibit the highest concentrations of adamantanes, diamantanes, and triamantanes, surpassing 10,000 ppm, 1900 ppm, and 210 ppm, respectively. The sample KL203 possessed the most abundant diamondoid concentration of 19137ppm among all the studied samples (Table 2). The chromatogram depicted in Fig. 2 presents a complete series of alkylated triamantanes as found in the KL203 condensate. It is noteworthy that abundant tetramantanes and alkylated tetramantanes were also detected in the Kela samples (not listed in Fig. 2). This implies these specific samples represent the most advanced maturity level within the Kuqa light oils. The concentrations of total adamantanes in samples TZ1, TZ3 and DB301 also exceed 9000 ppm, but their diamantane and triamantane contents are considerably lower than those in the Kela samples. In the remaining samples, the concentrations of total adamantanes, total diamantanes, and total triamantanes are less than 6000 ppm, 500 ppm, and 30 ppm, respectively (Fig. 3b,d). Among these, the Dabei and Dina samples exhibit relatively high diamondoid concentrations, followed by Bozi, Dibei, and Yangtake samples. The samples from Yingmai, Hongqi, Yaha, Wushi, Keshen, and Tudong regions display comparatively lower diamondoid concentrations. The lowest total diamondoid concentration is observed in the sample KS103, which is 141 ppm.

Certain specific and more easily detectable diamondoid compounds have been selected for widespread use. For instance, the classical plot of concentration of 4- + 3-MDs vs. C_29_ ααα 20R sterane, as initially proposed by Dahl et al.^12^, was served to estimate oil cracking and identify oil mixtures. Subsequently, numerous 4- + 3-MDs-based plots were constructed and used for the evaluation of thermal maturity, biodegradation level, oil-correlation, and evaporative fractionation^13,21,26,27^. As shown in Fig. 3e,f, the concentration trend of 4- + 3-MDs in samples from various regions is consistent with that of total diamantanes, i.e., Kela samples exhibit the highest abundance of 4- + 3-MDs, following by Dabei and Dina samples (Fig. 3). Given their contribution to total diamantanes up to 40 ~ 60% (Table 2), 4- + 3-MDs can serve as a substitute for the latter in relevant applications.

Moldowan et al.^21^ observed that the ratio of 1- + 2-Methyladamantanes (1- + 2-MAs) to (4- + 3-MDs) remains constant for a certain source regardless of oil cracking, suggesting an ideal indicator for source correlation. In theory, other ratios formulated as alkylated adamantanes to alkylated diamantanes, or alkylated diamantanes to alkylated triamantanes, should also share this similar feature. As shown in the plot of (1- + 2-MAs) vs. (4- + 3-MDs), the oils from Kuqa Depression fall on or near three trend lines (Fig. 3e,f). Similar trends are also found in the plots of As vs. Ds, Ds vs. Ts, and (4- + 3-MDs) vs. (9- + 8-MTs) (Fig. 3a–d,g,h). Notably, the samples exhibit unambiguous boundaries in the diagrams of Ds vs. Ts and (4- + 3-MDs) vs. (9- + 8-MTs), which may be attributed to the enhanced stability of diamantane and triamantane compounds when compared to adamantane ones. These samples are simply categorized into three groups: Group A comprises the samples from Keshen and Kela region (red samples); Group B contains the samples primarily from Bozi, Dabei, Dibei, Tudong, Yangtake, and Yaha regions (blue samples); while Group C includes the samples predominantly from Wushi, Dina, Yingmai, and Hongqi regions (black samples; Fig. 3). However, some samples with low diamondoid concentrations are difficult to be accurately classified without additional parameters.

As shown in Fig. 3, the slopes of linear fit decrease from Group C to Group A. For instance, the slopes (or average diamondoid ratios) for Group C, B, and A in the plot of (1- + 2-MAs) vs. (4- + 3-MDs) are 10.1, 7.2, and 2.5, respectively. The slope gradients are more pronounced in the plots of adamantanes vs. diamantanes and diamantanes vs. triamantanes (Fig. 3). In light of the significant deviations from the trend lines in these plots, the Tuzi samples are not assigned to any of the three identified groups and will be addressed subsequently. It is inferred that the light oils in the Kuqa Depression were derived from at least two different sets of source rocks. In our previous anhydrous pyrolysis experiments, we noted that the ratios of adamantane hydrocarbons to diamantane hydrocarbons (i.e., As/Ds, MAs/MDs) for type III kerogen were significantly higher than those for lacustrine type I and II kerogens across a broad maturity range^74^. Therefore, we proposed that the quality of source kerogen for these oils would improve from Group C to A (Fig. 3). Given that two main types of source rocks (i.e., lacustrine and coaly) have developed in the Kuqa Depression, we cautiously speculate that Group A is likely originated from lacustrine source rocks, Group C may be generated from coaly source rocks, while Group B could potentially be derived from transitional or coaly source rocks. Overall, concentrations and ratios of the diamondoids indicate that the Kuqa light oils have distinctly different sources and maturity ranges.

### Source facies identification for the Kuqa light oils

Diamondoids are believed to originate from the Lewis acid-catalyzed reactions of hydrocarbons and the cracking of macromolecular fractions, with the process likely beginning during early diagenesis and accompanying both the formation and cracking of oil^10,17,18^. As such, the abundance and distributions of diamondoids are thought to correlate with source facies (i.e., kerogen type and lithofacies). Several previous attempts had been made to establish the relationship between diamondoid parameters and source facies^20,25,75,76^. Walters et al.^27^ applied these proposed methods to determine the source facies of Eagle Ford oils and condensates, and found that these samples could be categorized into several source facies but did not align with the ranges defined in previous literature. Thus, Walters et al.^27^ suggested that diamondoids could only serve as auxiliary indicators, with the source facie needing to be confirmed through other more reliable methods.

**Fig. 4:**
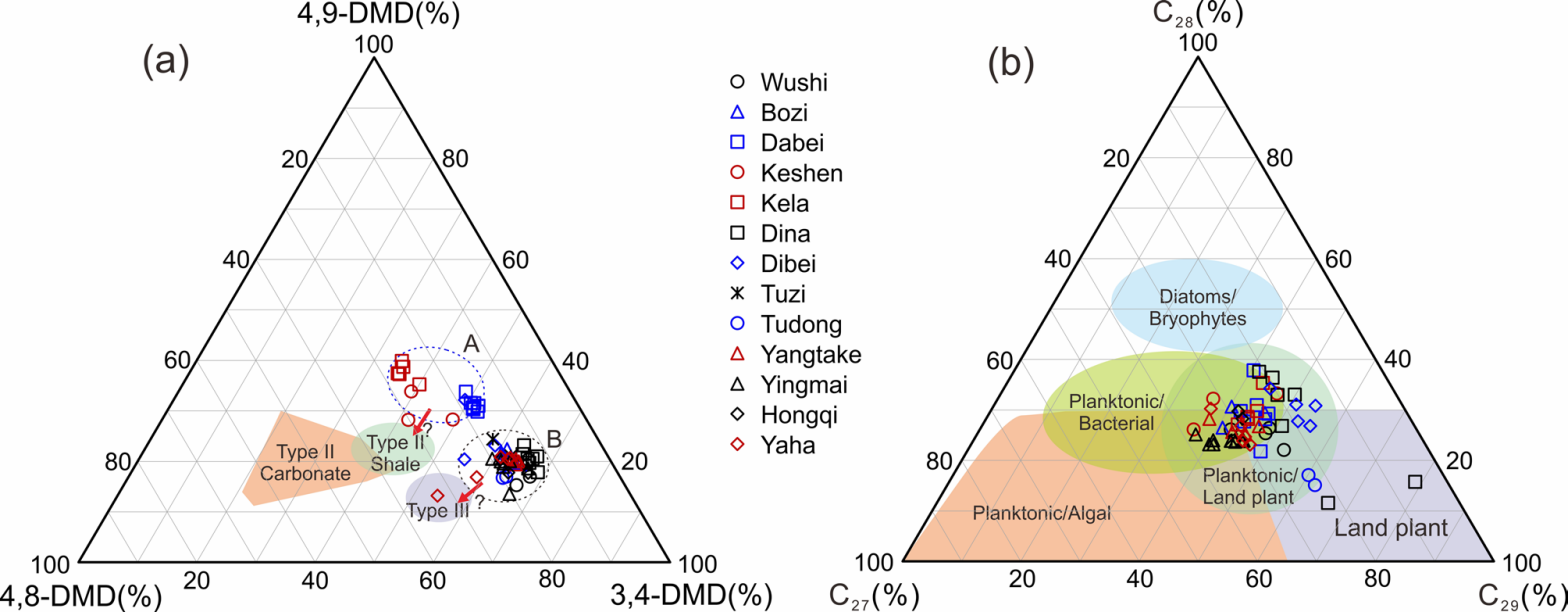
(**a**) Ternary plot of relative distribution of dimethydiamantane isomers (4,9-, 4,8-, and 3,4-DMD) for light oils from the Kuqa depression with source facies categories following Schulz et al.^20^. (**b**) Ternary plot of relative distribution of regular steranes (C_27_–C_29_) showing the types of organic matter input for light oils from the Kuqa depression (modified after Huang and Meinschein^78^.

Schulz et al.^20^ proposed several diamondoid indices (i.e., Dimethyldiamantane Index 1 and 2, Ethyladamantane Index) and a ternary diagram of the relative distribution of dimethyldiamantanes (4,9-, 4,8-, and 3,4-DMD) to define source facies fields, such as, carbonates, shales, and type III coals. These methods have been employed in numerous subsequent field studies. As shown in Fig. 4a, the light oils from the Kuqa Depression can distinctly classified into two regions (i.e., A and B) in the ternary plot, but almost all samples fall outside the ranges of source facies defined by Schulz et al.^20^. One plausible explanation is that the quantity of original samples used to delineate the source facies regions may be insufficient, indicating a need for boundary revisions as more samples become available. Another hypothesized reason is that these samples are at advanced mature levels, where dimethyldiamantane isomers have been converted into the more stable 4,9-DMD^21^, driving the samples to shift towards the upper of the ternary diagram.

Fang et al.^77^ also observed that the relative abundance of 4,9-DMD within its isomers increased with source rock maturation. This work demonstrated that the samples from the same source rock encompassing a wide maturity range (i.e., 1.69–2.52 EASY%Ro) exhibited clustered distribution within a restricted area in the ternary plot of relative distribution of dimethydiamantane isomers, with only one sample of 2.75 EASY%Ro showing distinct spatial separation from this cluster. As presented in Table 2, all calculated maturity values for the Kuqa samples are remain below 2.50 EASY%Ro. Furthermore, the maturity levels of samples from Regions B to A in Fig. 4a display gradual increases without abrupt transitions. These observations suggest that maturity effects do not account for the observed sample groupings in Fig. 4a, which should rather be attributed to source facies differences. Therefore, we conservatively assign the samples from Region A as lacustrine Type II shale facies and those from Region B as Type III/coal facies. However, it is challenging to distinguish these samples in the ternary plot of relative distribution of regular steranes, as almost of all samples exhibit a mixed organic matter input or type II facies, i.e., mixture of planktonic and bacterial or land plant, with two samples showing predominantly land plant input or type III facies (Fig. 4b). It seems that diamondoid parameters are proven more effective in distinguishing source facies compared to traditional biomarkers for these samples in this study, highlighting the promise application of diamondoids in high maturity stages.

**Fig. 5:**
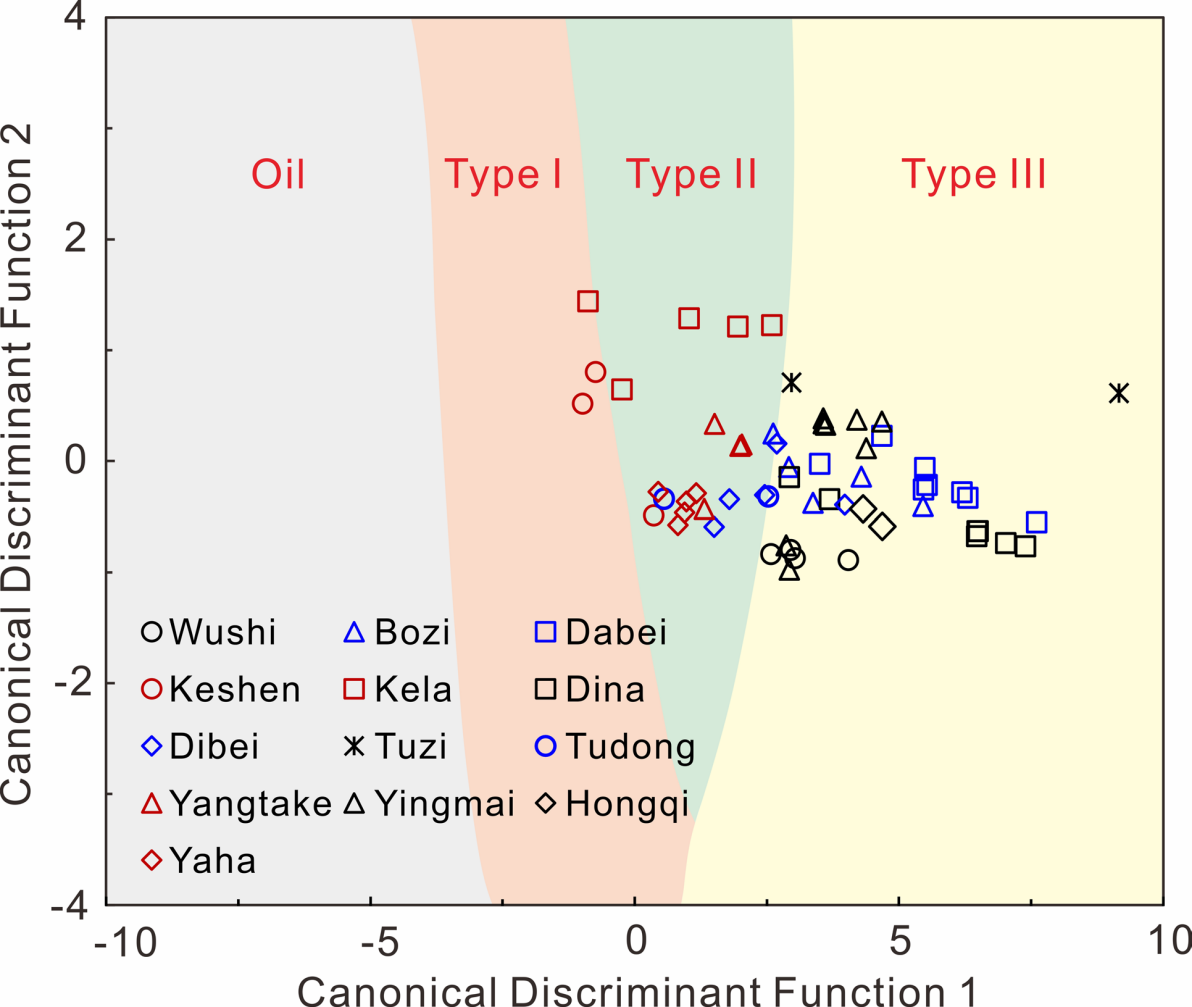
Discriminant plot showing source facies of light oils from Kuqa Depression with boundaries following Jiang et al.^65^. The field labeled ‘Oil’ refers to samples generated from the cracking of crude oil, while the fields labeled ‘Type I’, ‘Type II’, and ‘Type III’ represent samples derived from the thermal maturation of lacustrine Type I, Type II, and Type III kerogen, respectively.

In fact, each diamondoid parameter, including concentration, concentration ratio, and isomerization ratio, is affected by both source and maturity, and only applicable within a certain case. However, quantitative extended diamondoid analysis (QEDA) parameters represent an exception. This method, which involves comparing relative concentrations of higher diamondoids, enables determination of source rocks for any hydrocarbon fluids regardless of maturity^21,25^. Notably, triamantanes and higher diamondoids exhibit reduced detectability in most crude oil samples compared to adamantanes and diamantanes. Several dimethyldiamantane isomer ratios, initially proposed as source facies indicators by Schulz et al.^20^, have been subsequently utilized as maturity parameters in other works^15,74,79^. It means that it is difficult to exclude the influence of source when determining maturity using diamondoid index, and vice versa. As such, in our previous works, we utilized multivariate statistical analysis to extract source and maturity information from up to 50 diamondoid indices within diamondoid dataset obtained from thermal simulation experiments of different types of organic matter (i.e., lacustrine Type I, II, and III kerogens, and non-biodegraded oil)^65,74,79^. By integrating principal component analysis (PCA) and discriminant analysis (DA), we established a discriminant plot for source facies. Meanwhile, a maturity prediction function was also achieved by combining PCA and regression analysis (RA)^65^. Figure 5 displays the source facies of light oils from the Kuqa Depression determined by the constructed discriminant model. It suggests that the oils of Kela, Keshen, Yangtake, and Yaha regions are primarily deprived from lacustrine type I-II kerogens, while the samples collected from Wushi, Bozi, Dabei, Dina, Tuzi, Yingmai, and Hongqi regions are predominantly originated from type III or coaly source rocks. The oils from the Dibei and Tudong regions exhibit a mixed source of type II and III kerogens. This conclusion is largely consistent with that obtained by conventional biomarker method (Table 1). A notable discrepancy exists between the source classification of Dabei oils presented in Figs. 4a and 5. The Dabei oils depicted in Fig. 4a are interpreted as derived from lacustrine Type II shale facies, whereas these same oils fall distinctly within the Type III kerogen domain in Fig. 5. This observed inconsistency likely stems from the diamondoid parameters utilized in Fig. 4a potentially reflecting not only source facies but also thermal maturity variations. In contrast, the source attributions shown in Fig. 5 exhibit greater reliability through application of multivariate statistical analysis that effectively mitigates thermal maturity effects. Additionally, we acknowledge that the source assignments derived from the PCA–DA diamondoid discriminant model (Fig. 5) represent probabilistic classifications rather than definitive source determinations, with an accuracy of 86% for cross-validated grouped cases as documented in the work of Jiang et al.^65^.

### Maturity evaluation for the Kuqa light oils

The diamondoid isomerization ratios were constructed based on the more stability of isomer with alkyl-substitution on bridgehead position comparing to other position. For example, the MAI and MDI, proposed by Chen et al.^11^, were the most widely used diamondoid maturity indicators. Subsequently, more diamondoid isomerization indices were established based on the above principle, including EAI, DMAI-1, DMAI-2, TMAI-1, TMAI-2, DMAI-1, DMAI-2, and MTI^15,16^. Numerous works demonstrated that these isomerization ratios generally increased with increasing maturity at advanced stages, i.e., Ro > 1.3%^16–19,74,79^. Van der Ploeg et al.^80^ proposed a novel maturity parameter designated as adamantane index (AI), calculated from the relative abundances of adamantane and its suspected precursor perhydrotriquinacene. The parameter demonstrated a strong linear correlation with thermal maturity across an extensive maturity range (i.e., 0.6–1.6%Ro), independent of source rocks.

**Fig. 6:**
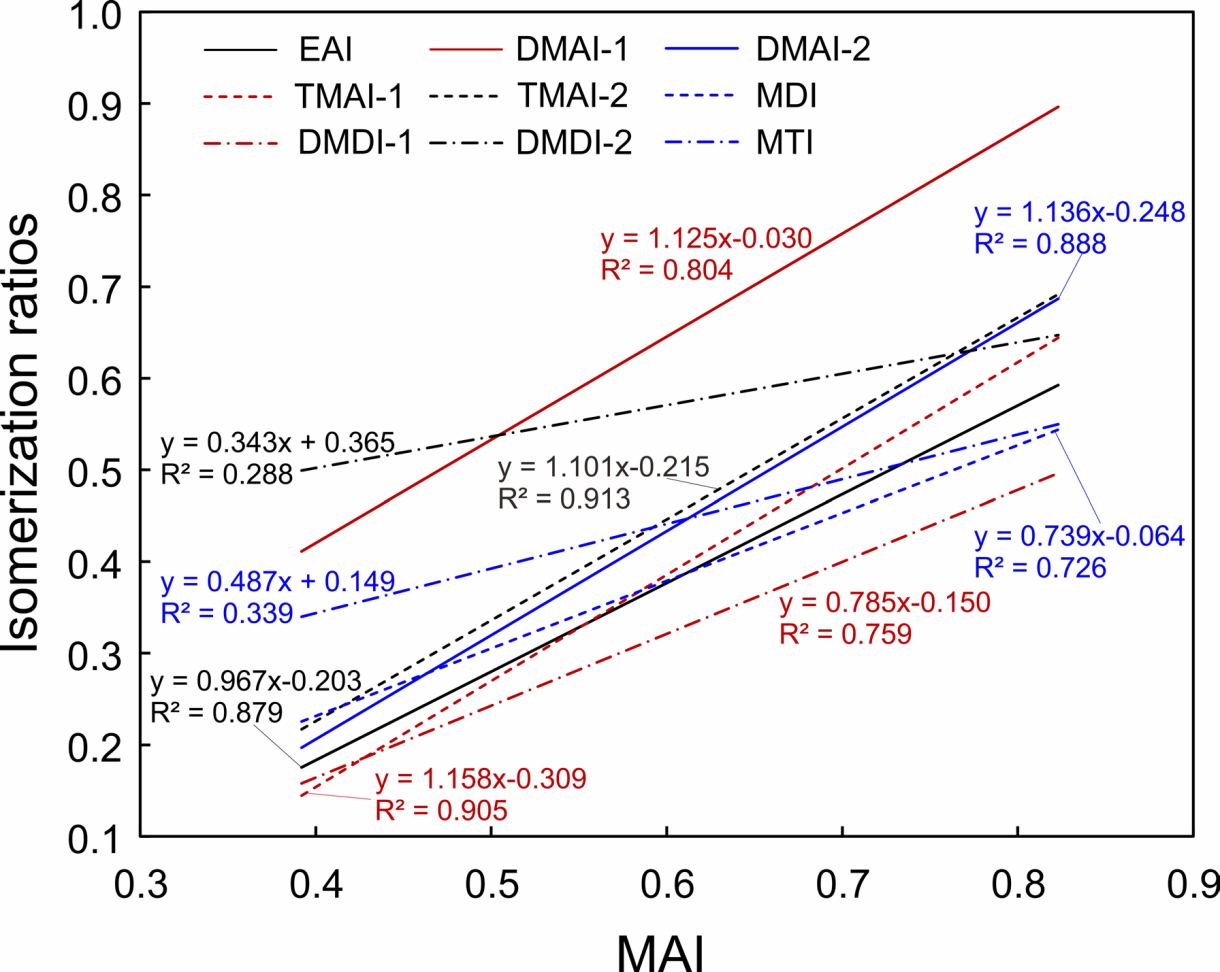
Plot showing the linear fitting relationships between MAI and other diamondoid isomerization ratios for the light oils from Kuqa Depression.

As illustrated in Fig. 6, MAI presents good positive linear correlations with other diamondoid isomerization ratios except DMDI-2 and MTI, with the R^2^ values of these fitted lines above 0.72. Conversely, the R^2^ values for the relationships between MAI and both DMDI-2 and MTI are less than 0.35, indicating their weak linear correlations. It is roughly consistent with the features observed by Huang et al.^57^. In that study, they observed that diamondoid isomerization ratios exhibited good positive relationships with each other and with other maturity indices (i.e., dryness ratios), suggesting that these ratios as effective maturity parameters. And they attributed the relative poorer correlations of MAI to EAI and DMDI-2 to the influence of source facies on EAI, DMDI-1, and DMDI-2, as proposed by Schulz et al.^20^. Our prior research demonstrated that diamondoid isomerization ratios were insensitive to source facies, exhibiting nearly consistent values at lower maturity (i.e., EASY%Ro < 1.7) and increasing trends at higher maturity (i.e., EASY%Ro > 1.7) across various kerogen types (i.e., lacustrine I, II, and III kerogens)^74^. We speculate that the aforementioned weak correlations may be attributed to certain samples not yet reaching the maturity threshold where diamondoid isomerization ratios exhibit pronounced linear relationships with thermal maturity, particularly those ratios (i.e., DMDI-2, MTI) demonstrating maturity dependence exclusively under advanced maturity level^16,63,74^.

**Fig. 7:**
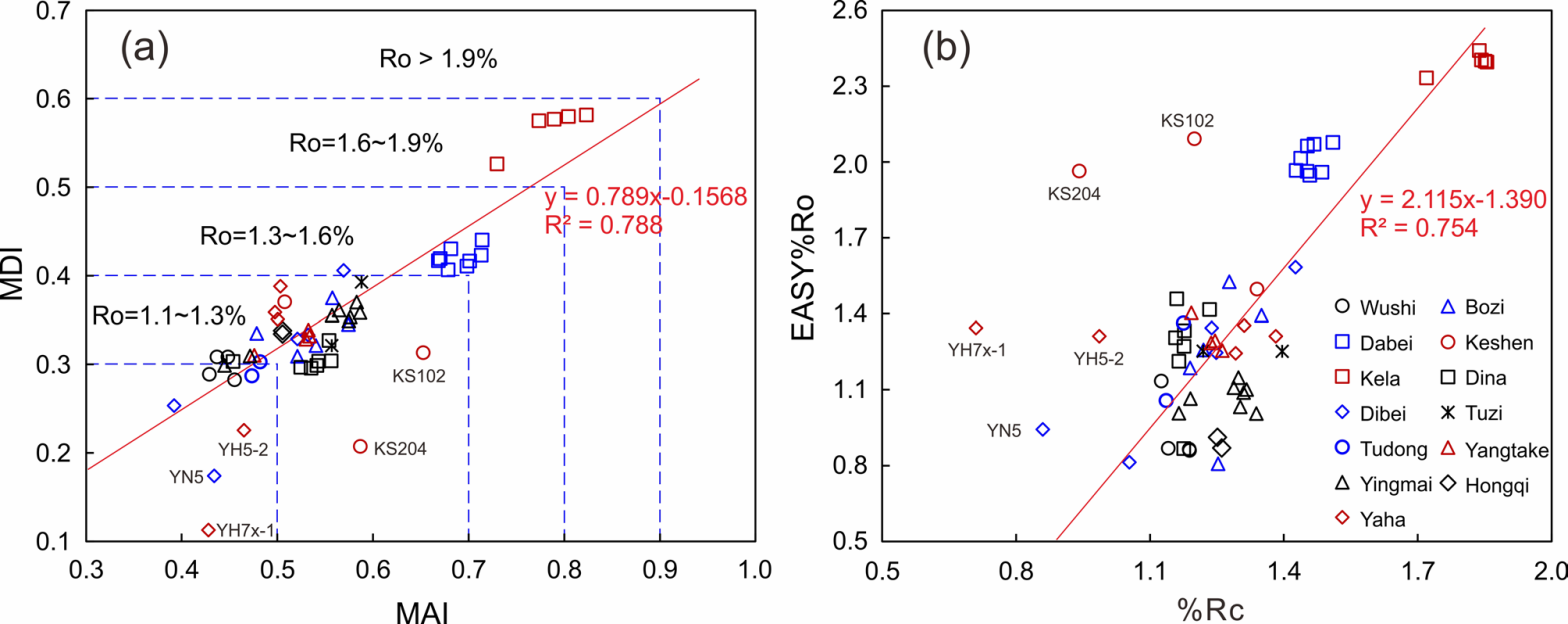
(**a**) Plot of methyladamantane index (MAI) vs. methyldiamantane index (MDI) with maturity ranges proposed by Chen et al.^11^. (**b**) Plot of %Rc vs. EASY%Ro for the oils from Kuqa Depression. See Table 2 and text for the definition of %Rc and EASY%Ro. Five samples (i.e., KS102, KS204, YN5, YH5-2, and YH7x-1) were excluded from the fitted lines.

The maturity of oils in the Kuqa Depression are also quantitatively calculated based on the empirical formula of diamondoid indices. As displayed in Table 2, the maturity range of the studied samples is 0.81–2.44 EASY%Ro (or 0.71–1.85%Rc). The difference between the two maturity ranges is mainly attributed to the different maturity standards. %Rc has been calibrated to vitrinite reflectance %VRo, which was derived from measured bitumen reflectance using an empirical correlation^11^. EASY%Ro provides a maturity scale based on the method proposed by Sweeney and Burnham^66^. Burnham and Sweeney^81^ also suggested that calculated EASY%Ro and measured %VRo values generally agree within 0.1 at low rank and 0.4 at high rank. As shown in Fig. 7, good linear correlations are observed between MAI and MDI, as well as %Rc and EASY%Ro, with R^2^ values of 0.788 and 0.761, respectively, when five samples (i.e., KS102, KS204, YN5, YH5-2, and YH7x-1) that significantly deviated from the fitted lines are excluded. Here we only analyze the EASY%Ro maturity, as the prediction method can effectively avoid the limitations of single diamondoid index. Detailedly, the samples from the Kela exhibit the highest maturity within all samples from Kuqa Depression, ranging from 2.3 to 2.5 EASY%Ro. It is followed by the Dabei and Keshen samples, which present a maturity of 1.5–2.1 EASY%Ro. While the samples from Bozi, Dina, Dibei, Tuzi, Tudong, Yangtake, and Yaha regions primarily share a similar maturity level of 1.1–1.5 EASY%Ro. Notably, the samples from Wushi, Yingmai, and Hongqi areas display the lowest maturity, with the range of 0.8–1.2 EASY%Ro. We review the diamondoid isomerization indices of the above mentioned five samples (i.e., KS102, KS204, YN5, YH5-2, and YH7x-1) and find that these samples possess significantly lower MDI value but similar values for most of other isomerization indices when comparing with samples from the same or adjacent regions (Table 2). It may be attributed to the uncertainty of MDI value that caused by low diamantane abundance or secondary alteration.

Some diamondoid concentrations (i.e., 4- + 3-MDs) and compositional ratios (i.e., MAs/MDs, A/MAs, As/Ds) are also employed as maturity indices, but they often exhibit source dependency, rendering them unsuitable for samples with diverse source facies^17,27,74,82^. This characteristic is also observed in samples from Kuqa Depression (Fig. 3). Consequently, diamondoid abundance and compositional ratios are recommended as maturity parameters exclusively for samples from a homogenous source and are not discussed in this paper. Therefore, the EASY%Ro value, as predicted by the maturity function established through multivariate statistical analysis of numerous diamondoid indices, is effective for assessing the maturity of the light oils in the Kuqa depression.

### Formation mechanism of anomalous abundance and distribution of diamondoids

Based on the preceding discussion, the genetic origins of light oils within the Kuqa Depression can be systematically elucidated. The light oils from the Kela field, characterized by exceptionally high diamondoid concentrations (total diamondoids > 10,000 ppm, except for KL3), are interpreted to originate from lacustrine source rocks at high to over-mature stages (i.e., 2.3–2.5 EASY%Ro). In contrast, samples from the Keshen region display comparatively low diamondoid contents (i.e., total diamondoids < 1100 ppm) despite being speculated to originate from mature to high-maturity lacustrine shales (i.e., 1.5–2.1 EASY%Ro), exhibiting an inconsistent correlation between diamondoid concentration and thermal maturity level. Samples from the Yangtake and Yaha areas are proposed to source from lacustrine shales with a maturity of 1.2–1.4 EASY%Ro. The remaining samples are predominantly considered to originate from type III or coaly source rocks, with the exception of YN2-1, YN5, and TD201, which are inferred to derive from lacustrine shales. Notably, the light oils from the Dabei field are derived from highly mature coaly source rocks (i.e., 1.9–2.1 EASY%Ro). The majority of coal-derived oils from Bozi, Dina, Dibei, Tuzi, and Tudong regions are generated at a maturity level of 1.1–1.5 EASY%Ro. Conversely, samples from Wushi, Yingmai, and Hongqi areas are originated from coaly source rocks with relatively low maturity levels (i.e., 0.8–1.2 EASY%Ro).

**Fig. 8:**
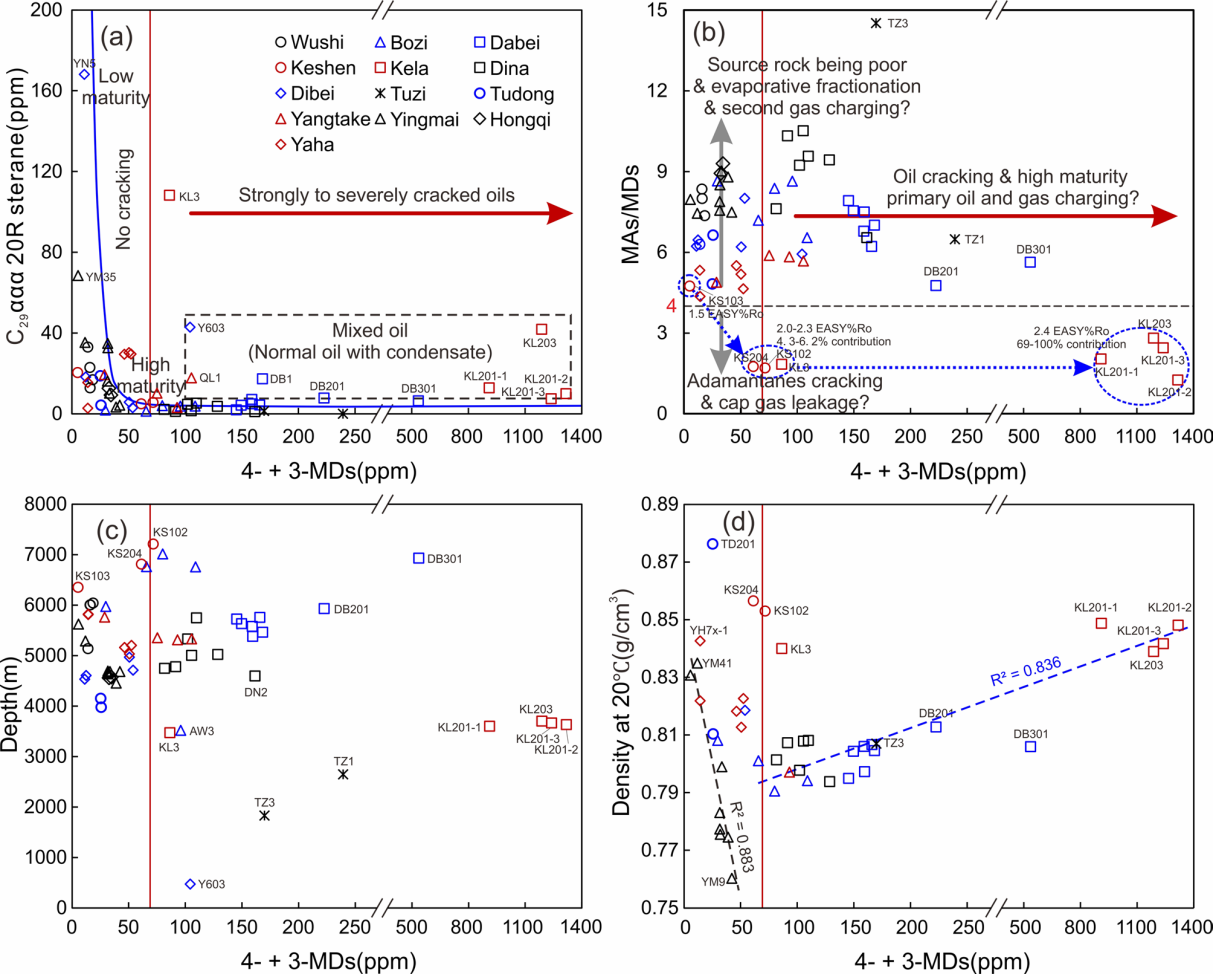
Plots of (**a**) 4- +3-MDs vs. C_29_ ααα20R sterane concentration, (**b**) 4- +3-MDs vs. MAs/MDs, (**c**) 4- +3-MDs vs. Depth, and (**d**) 4- +3-MDs vs. Density at 20 ℃ for the light oils from Kuqa Depression. The diamondoid baseline (red line) in the figure is included solely for schematic illustration. The percentage near the circles in Fig. 8b refers to the contribution of highly mature condensate endmember.

Nevertheless, some samples display anomalous abundance and distribution of diamondoids, necessitating further investigation. These include, but not limited to, samples from the Dabei, Kela and Tuzi areas, which hold excessively high diamondoid concentrations, and samples from Keshen that present low diamondoid concentrations are inconsistent with their high maturity level. The abundance and distribution of diamondoids are the comprehensive results of various factors, such as source facies, thermal maturity, expulsion efficiency, and secondary processes. As shown in Fig. 8a, the classic plot of concentrations of 4- + 3-MDs vs. C_29_ ααα20R sterane illustrates that the light oils from Dabei and Kela regions, along with several samples from other areas (i.e., QL1, Y603), demonstrate a mixed origin of normal maturity oil with highly mature condensates. As showing in Fig. 5, all the oils studied in this paper are inferred to derive from kerogen maturation rather than crude oil cracking, the high maturity endmembers for these mixed samples would represent oils generated from the maturation of kerogens at advanced stages. It is important to note that the high abundance of diamondoids does not necessarily originate from oil cracking; extracts of highly mature coals and shales also contain elevated diamondoid concentrations (i.e., 4- + 3-MDs > 200 ppm)^13^.

For these lacustrine oil samples from the Kela and Keshen areas, the KS103 sample, which have relative lower diamondoid abundance and maturity (i.e., 1.5 EASY%Ro), could be served as the mature crude oil endmember. While the KL201-2 sample, which has high diamondoid concentrations and maturity (i.e., 2.44 EASY%Ro), would be regarded as the higher maturity condensate endmember. The above inference is reasonable. Guo et al.^60^ suggested that light oils from the Kela-2 gas field were mainly derived from the terrestrial mudstones in the Triassic Huangshanjie (T_3_h) with a maturity level of 1.6–1.8%Ro, while gas in this field was generated at a thermal maturity level of 2.1–2.5%Ro. In fact, the %Ro for gas here should be EASY%Ro as the gas maturity was calculated based on the model of gas carbon isotopes proposed by Berner and Faber^83^. Thus, the high maturity condensates are associated with gas generation from highly mature to over-mature source rocks. Based on the concentrations of 4- + 3-MDs, a preliminary estimation indicates that the contribution of high maturity endmember to the samples from the Kela and Keshen areas ranges between 4 and 100% (Fig. 8b). The mixing of small amount of high maturity condensate could obviously elevate the diamondoid-based maturity of the mixed oil. For example, the samples of KS102, KS204, and KL3 containing 4.3–6.2% of the high maturity condensates show a maturity range of 2.0–2.3 EASY%Ro. Thus, a simple and plausible scenario can be speculated: the reservoirs here were initially charged with oils derived from the Huangshanjie lacustrine mudstones at late mature stage (i.e., 1.5 EASY%Ro), followed by a secondary charge of highly mature oil and gas generated at higher maturity stages (i.e., 2.1–2.5 EASY%Ro). Given that the deep reservoirs (i.e., KS102, KS103, and KS204) were firstly saturated with mature crude oil, the subsequent highly mature oil and gas predominantly occupied the shallower reservoirs, resulting in a great presence of higher maturity condensate in these shallow reservoirs (i.e., KL201-1, KL201-2, KL201-3, and KL203; Fig. 8b, c).

Addition, the samples from Kela and Keshen fields (except for KS103) have the lowest MAs/MDs ratios (i.e., 1.2–2.5) among these studied samples. Huang et al.^56^ suggested that condensates that have higher 4- +3-MDs content and lower MAs/MDs ratios in the range of 2–4 have received primary gas charges from deep post mature source rocks, while condensate that have MAs/MDs ratios < 2 would have experienced cap gas leakage. An alternative interpretation is that the primary condensates of higher maturity inherently possess notably low MAs/MDs ratios due to the cracking of adamantanes during advanced maturity stages. In our previous research, we observed that the ratios of alkylated adamantanes to alkylated diamantanes elevate with increasing maturity when < 1.6 EASY%Ro and descend with increasing maturity when > 1.6 EASY%Ro^74^. Consequently, the MAs/MDs ratio for type III kerogen fell below 2 at 2.4 EASY%Ro, and this maturity were even lower for lacustrine type I and II kerogens^74^. Therefore, our explanation is reasonable; however, the possibility of cap gas leakage cannot be ruled out and requires further evidence.

The TZ1 and TZ3 samples from the Tuzi area exhibit high concentrations of diamondoids (i.e., total diamondoids > 9000 ppm) but low contents of biomarkers, suggesting a single origin of highly mature source rocks (Fig. 8a). The C_29_ ααα20R sterane is undetectable in sample TZ1 and presents only trace amount (i.e., 1.3 ppm) in sample TZ3. But their predicted maturity levels are relatively lower, i.e., 1.25 EASY%Ro, which do not match with their high diamondoid contents and lower biomarker contents. This implies that both TZ1 and TZ3 possibly experienced secondary alteration. Sample TZ3 has the highest MAs/MDs ratio of 14.5 among all the studied samples, while sample TZ1 has a MAs/MDs ratio of 6.5 (Fig. 8b). Huang et al.^57^ suggested that oils charged by secondary gases from the cap gas leakage of other deeper reservoirs would exhibit high MAs/MDs ratios. The shallower sample TZ3 have higher abundance of adamantanes but lower abundance of diamantanes and triamantanes compared with the deeper sample TZ1 (Table 2; Fig. 8). This characteristic may also be induced by evaporative (or migration) fractionation, which leads to the enrichment of more soluble adamantanes and the depletion of less soluble diamantanes and triamantanes in the light fraction when compared to the original oil and residual fractions^38^. Thus, both samples (TZ1 and TZ3) may be the migrating condensate from deeper reservoirs, and the shallower one (TZ3) exhibits higher MAs/MDs ratio and greater degree of fractionation. This suggests that potential oil and gas resources exist in deep strata of the Tuzi area.

In addition, some other interesting phenomena are observed among these samples. For examples, the density of oil samples initially decreases and subsequently increases in correlation with the concentration increasing of 4- + 3-MDs, with a concentration boundary of 50–60 ppm (Fig. 8d). The density of these samples tends to decrease with increasing maturity at relatively low maturity stages due to the degradation of unstable macromolecular components (i.e., NSO compounds and asphaltenes), also suggesting a primary generation mechanism for these light oils. Conversely, at advanced maturity stages or oil cracking stages, the density of the residual oil increased with maturity can be attributed to the concentration increasing of stable macromolecular compounds (i.e., highly polymeric bitumen and aromatic fractions) or phase fractionation, indicating a primary or secondary generation mechanism for these light oils, which need further investigation.

## Conclusions

1. The light oils within the Kuqa Depression exhibit variations in diamondoid concentrations, with total diamondoid of 141–19,137 ppm. The highest total diamondoid concentrations are evident in the light oils from the Kela and Tuzi regions (> 9000 ppm), followed by those from the Dabei and Dina regions, which mainly fall within the 4000–6000 ppm range. The oils from the Bozi, Dibei, and Yangtake regions possess total diamondoid concentrations that primarily range from 1500 to 4000 ppm, whereas samples from the Yingmai, Hongqi, Yaha, Wushi, Keshen, and Tudong regions present notably lower diamondoid concentrations (< 2000 ppm).
2. The Kuqa oils are identified to derive from two distinct types of source rocks: lacustrine shales and coals, as determined by a source facies discriminant model developed via multivariate statistical analysis on a series of diamondoid indices. Specifically, the oils from the Kela, Keshen, Yangtake, and Yaha regions are originated from lacustrine shales, while the remaining studied samples of Kuqa Depression are predominantly derived from coaly source rocks.
3. Our thermal maturity quantitative prediction model indicates that the maturity of the Kuqa oils varies between 0.81 and 2.44 EASY%Ro. The Kela light oils display the highest maturity (2.3–2.5 EASY%Ro), followed by the Dabei and Keshen light oils (1.5–2.1 EASY%Ro). The oils from the Bozi, Dina, Dibei, Tuzi, Tudong, Yangtake, and Yaha regions largely exhibit a comparable maturity level, ranging from 1.1 to 1.5 EASY%Ro. The lowest maturity is observed in the samples from the Wushi, Yingmai, and Hongqi areas, which range from 0.8 to 1.2 EASY%Ro.
4. The Keshen and Kela lacustrine light oils are mixing origin of mature crude oil (i.e., 1.5 EASY%Ro) with highly mature condensates (i.e., 2.1–2.5 EASY%Ro). The Kela light oils, characterized by high maturity and high diamondoid concentrations, indicate a significant contribution from highly mature condensates (69–100%) that generated from the maturation of kerogens at advanced stages. While the Keshen oils, also exhibiting high maturity but lower diamondoid concentrations, have low contents of highly mature condensates (0–6.2%). The Tuzi coal-derived oils, presenting high adamantane concentrations but unmatched relative lower diamantane concentrations, are speculated to be the products of evaporative (or migration) fractionation during upward migration from deeper reservoirs. Thus, the diamondoid parameters are effective in determining the source facies and thermal maturity, as well as formation mechanism, for the light oils from the Kuqa Depression.

## Data Availability

The datasets generated and/or analysed during the current study are available from the corresponding author on reasonable request.

## References

[1] Peters, K. E., David, J. C. & Marek, K. An overview of basin and petroleum system modeling: Definitions and concepts. In *Basin Modeling: New Horizons in Research and Applications: AAPG Hedberg Series*, vol. 4 (eds. Peters, K. E. et al.) 1–16 (2012).

[2] Thompson, K. F. M. Fractionated aromatic petroleums and the generation of gas condensates. *Org. Geochem.* **11**, 573–590 (1987).

[3] Meulbroek, P., Cathles, L. & Whelan, J. Phase fractionation at South Eugene Island block 330. *Org. Geochem.* **29**, 223–239 (1998).

[4] van Graas, G. W., Gilje, A. E., Isom, T. P. & Tau, L. A. The effects of phase fractionation on the composition of oils, condensates and gases. *Org. Geochem.* **31**, 1419–1439 (2000).

[5] Losh, S., Cathles, L. & Meulbroek, P. Gas washing of oil along a regional transect, offshore Louisiana. *Org. Geochem.* **33**, 655–663 (2002).

[6] Losh, S. & Cathles, L. Phase fractionation and oil-condensate mass balance in the South marsh Island block 208–239 area, offshore Louisiana. *Mar. Pet. Geol.* **27**, 467–475 (2010).

[7] Zhang, S. C. et al. Geochemistry of palaeozoic marine petroleum from the Tarim Basin, NW china: part 3. Thermal cracking of liquid hydrocarbons and gas washing as the major mechanisms for deep gas condensate accumulations. *Org. Geochem.* **42**, 1394–1410 (2011).

[8] Peng, P. & Jia, C. Z. Evolution of deep source rock and resource potential of primary light oil and condensate. *Acta Petrolei Sinica*. **42**, 1543–1555 (2021).

[9] Zhang, S. C., Su, J., Zhang, B., Wang, X. M. & He, K. Genetic mechanism and controlling factors of deep marine light oil and condensate oil in Tarim basin. *Acta Petrolei Sinica*. **42**, 1566–1580 (2021).

[10] Wingert, W. S. GC-MS analysis of diamondoid hydrocarbons in smackover petroleums. *Fuel* **71**, 37–43 (1992).

[11] Chen, J. H., Fu, J. M., Sheng, G. Y., Liu, D. H. & Zhang, J. J. Diamondoid hydrocarbon ratios: novel maturity indices for highly mature crude oils. *Org. Geochem.* **25**, 179–190 (1996).

[12] Dahl, J. E. et al. Diamondoid hydrocarbons as indicators of natural oil cracking. *Nature* **399**, 54–57 (1999).

[13] Wei, Z. B., Moldowan, J. M., Jarvie, D. M. & Hill, R. The fate of diamondoids in coals and sedimentary rocks. *Geology* **34**, 1013–1016 (2006).

[14] Li, J. G., Philp, P. & Cui, M. Z. Methyl Diamantane index (MDI) as a maturity parameter for lower palaeozoic carbonate rocks at high maturity and overmaturity. *Org. Geochem.* **31**, 267–272 (2000).

[15] Zhang, S. C., Huang, H. P., Xiao, Z. Y. & Liang, D. G. Geochemistry of palaeozoic marine petroleum from the Tarim Basin, NW China. Part 2: maturity assessment. *Org. Geochem.* **36**, 1215–1225 (2005).

[16] Wei, Z. B. et al. Diamondoid hydrocarbons as a molecular proxy for thermal maturity and oil cracking: geochemical models from hydrous pyrolysis. *Org. Geochem.* **38**, 227–249 (2007).

[17] Wei, Z. B., Moldowan, J. M. & Paytan, A. Diamondoids and molecular biomarkers generated from modern sediments in the absence and presence of minerals during hydrous pyrolysis. *Org. Geochem.* **37**, 891–911 (2006).

[18] Fang, C. C. et al. The origin and evolution of Adamantanes and Diamantanes in petroleum. *Geochim. Cosmochim. Acta*. **120**, 109–120 (2013).

[19] Li, Y. et al. Origin of Adamantanes and Diamantanes in marine source rock. *Energy Fuels*. **29**, 8188–8194 (2015).

[20] Schulz, L. K., Wilhelms, A., Rein, E. & Steen, A. S. Application of diamondoids to distinguish source rock facies. *Org. Geochem.* **32**, 365–375 (2001).

[21] Moldowan, J. M., Dahl, J., Zinniker, D. & Barbanti, S. M. Underutilized advanced geochemical technologies for oil and gas exploration and production-1. The diamondoids. *J. Pet. Sci. Eng.* **126**, 87–96 (2015).

[22] Li, Y. et al. The application of diamondoid indices in the Tarim oils. *AAPG Bull.* **102**, 267–291 (2018).

[23] Esegbue, O., Jones, D. M., van Bergen, P. F. & Kolonic, S. Quantitative diamondoid analysis indicates oil cosourcing from a deep petroleum system onshore Niger delta basin. *AAPG Bull.* **104**, 1231–1259 (2020).

[24] Spaak, G. et al. Identifying multiple sources of petroleum fluids in browse basin accumulations using diamondoids and semi-volatile aromatic compounds. *Mar. Pet. Geol.* **113**, 104091 (2020).

[25] Atwah, I., Moldowan, J. M., Koskella, D. & Dahl, J. Application of higher diamondoids in hydrocarbon mudrock systems. *Fuel* **284**, 118994 (2021).

[26] Forkner, R., Fildani, A., Ochoa, J. & Moldowan, J. M. Linking source rock to expelled hydrocarbons using diamondoids: an integrated approach from the Northern Gulf of Mexico. *J. Pet. Sci. Eng.* **196**, 108015 (2021).

[27] Walters, C. C., Sun, X. & Zhang, T. Geochemistry of oils and condensates from the lower eagle Ford formation, South Texas. Part 4: diamondoids. *Mar. Pet. Geol.* **154**, 106308 (2023).

[28] Grice, K., Alexander, R. & Kagi, R. I. Diamondoid hydrocarbon ratios as indicators of biodegradation in Australian crude oils. *Org. Geochem.* **31**, 67–73 (2000).

[29] Wei, Z. B., Moldowan, J. M., Peters, K. E., Wang, Y. & Xiang, W. The abundance and distribution of diamondoids in biodegraded oils from the San Joaquin valley: implications for biodegradation of diamondoids in petroleum reservoirs. *Org. Geochem.* **38**, 1910–1926 (2007).

[30] Cheng, X., Hou, D. J. & Xu, C. G. The effect of biodegradation on Adamantanes in reservoired crude oils from the Bohai Bay Basin, China. *Org. Geochem.* **123**, 38–43 (2018).

[31] Chang, X. et al. Biodegradation levels of oils from the chepaizi Uplift, Junggar basin (NW China) evaluated by a full-range biodegradation index as constrained by adamantane, Diamantane homologs and carboxylic acids. *Mar. Pet. Geol.* **146**, 105939 (2022).

[32] Sassen, R. & Post, P. Enrichment of diamondoids and ^13^C in condensate from Hudson Canyon, US Atlantic. *Org. Geochem.* **39**, 147–151 (2008).

[33] Zhu, G. Y. et al. Diamondoids as tracers of late gas charge in oil reservoirs: example from the Tazhong area, Tarim Basin, China. *Fuel* **253**, 998–1017 (2019).

[34] Chai, Z. & Chen, Z. Biomarkers, light hydrocarbons, and diamondoids of petroleum in deep reservoirs of the Southeast Tabei Uplift, Tarim basin: implication for its origin, alteration, and charging direction. *Mar. Pet. Geol.* **147**, 106019 (2023).

[35] Atwah, I., Azzouni, A. & Alalawi, W. Time-lapse diamondoid analysis in unconventional reservoirs: A new frontier in production monitoring. *SPE/AAPG/SEG Unconventional Resources Technology Conference (URTeC)*, D021S037R003 (2024).

[36] AlSaif, M. et al. Diamondoids, biomarkers, and chemofacies: uniting insights on hydrocarbon maturation and migration in the Cline Shale, Midland basin. *Org. Geochem.* **203**, 104953 (2025).

[37] Atwah, I., Mohammadi, S., Moldowan, J. M. & Dahl, J. Episodic hydrocarbon charge in tight Mississippian reservoirs of central Oklahoma, USA: insights from oil inclusion geochemistry. *Mar. Pet. Geol.* **123**, 104742 (2021).

[38] Chakhmakhchev, A., Sanderson, J., Pearson, C. & Davidson, N. Compositional changes of diamondoid distributions caused by simulated evaporative fractionation. *Org. Geochem.* **113**, 224–228 (2017).

[39] Zhu, G. Y. et al. Deepest oil in asia: characteristics of petroleum system in the Tarim basin, China. *J. Pet. Sci. Eng.* **199**, 108246 (2021).

[40] Qi, Y., Sun, P., Cai, C., Wang, D. & Peng, Y. Phase fractionation controlling regional distribution of diamondoids: A case study from the Halahatang oil field, Tarim Basin, China. *Mar. Pet. Geol.* **140**, 105674 (2022).

[41] Liang, D. G., Zhang, S. C., Chen, J., Wang, F. & Wang, P. Organic geochemistry of oil and gas in the Kuqa depression, Tarim Basin, NW China. *Org. Geochem.* **34**, 873–888 (2003).

[42] Zhu, G. Y. et al. Geochemistry, origin and accumulation of continental condensate in the ultra-deep-buried cretaceous sandstone reservoir, Kuqa Depression, Tarim Basin, China. *Mar. Pet. Geol.* **65**, 103–113 (2015).

[43] Zhu, G. Y. et al. The complexity, secondary geochemical process, genetic mechanism and distribution prediction of deep marine oil and gas in the Tarim Basin, China. *Earth-Sci. Rev.* **198**, 102930 (2019).

[44] Yang, H. J. et al. Accumulation conditions, key exploration and development technologies for Keshen gas field in Tarim basin. *Acta Petrolei Sinica*. **42**, 399–414 (2021).

[45] Wang, Q. H. et al. Major breakthrough and exploration significance of well Ketan 1 in Kuqa Depression, Tarim basin. *China Pet. Explor.* **28**, 1–10 (2023).

[46] Zhao, W. Z. et al. Gas systems in the Kuche depression of the Tarim basin: source rock distributions, generation kinetics and gas accumulation history. *Org. Geochem.* **36**, 1583–1601 (2005).

[47] Bao, J., Zhu, C., Zhang, Q., Li, M. & Lu, Y. Geochemical characteristics of crude oil from frontal uplift in Kuqa depression. *J. Oil Gas Tech.* **29**, 40–44 (2007).

[48] Zhang, S. C., Zhang, B., Zhu, G., Wang, H. & Li, Z. Geochemical evidence for coal-derived hydrocarbons and their charge history in the Dabei gas Field, Kuqa thrust Belt, Tarim Basin, NW China. *Mar. Pet. Geol.* **28**, 1364–1375 (2011).

[49] Bao, J., Zhu, C. & Shen, X. Study on diamondoids and genetic mechanism of condensates from the Kela 2 structure in the Kuche depression. *Nat. Gas Geosci.* **29**, 1217–1230 (2018).

[50] Deng, H. et al. Geochemical characteristics of light crude oils/condensates from the Kelasu structural belt of the Kuqa depression in the Tarim Basin, Northwest China. *Geochimica* **53**, 668–680 (2024).

[51] Ji, H., Huang, G., Cheng, D. & Xu, S. Geochemical application of light hydrocarbons in Kuqa depression of Tarim basin: case study of Dawanqi-Dabei areas. *Nat. Gas Geosci.* **28**, 965–974 (2017).

[52] Zhu, G. Y. et al. Composition and origin of molecular compounds in the condensate oils of the Dabei gas field, Tarim Basin, NW China. *Pet. Explor. Dev.* **46**, 482–495 (2019).

[53] Chai, Z. et al. Light hydrocarbons and diamondoids of light oils in deep reservoirs of shuntuoguole low Uplift, Tarim basin: implication for the evaluation on thermal maturity, secondary alteration and source characteristics. *Mar. Pet. Geol.* **117**, 104388 (2020).

[54] Chai, Z. et al. Light hydrocarbons and diamondoids in deep oil from Tabei of Tarim basin: implications on petroleum alteration and mixing. *Mar. Pet. Geol.* **138**, 105565 (2022).

[55] Zhou, C. et al. Oil maturities, mixing and charging episodes in the cratonic regions of the Tarim Basin, NW china: insight from biomarker and diamondoid concentrations and oil bulk properties. *Mar. Pet. Geol.* **126**, 104903 (2021).

[56] Huang, W. et al. Diamondoid fractionation and implications for the Kekeya condensate field in the Southwestern depression of the Tarim Basin, NW China. *Mar. Pet. Geol.* **138**, 105551 (2022).

[57] Huang, W., Zhang, H., Xiao, Z., Yu, S. & Pan, C. Generation, expulsion and accumulation of diamondoids, aromatic components and gaseous hydrocarbons for gas fields in Kuqa depression of the Tarim Basin, NW China. *Mar. Pet. Geol.* **145**, 105893 (2022).

[58] Qiao, R., Li, M., Zhang, D. & Xiao, H. Distribution and origin of higher diamondoids in the ultra-deep paleozoic condensates of the Shunbei oilfield in the Tarim Basin, NW China. *Org. Geochem.* **197**, 104883 (2024).

[59] Qiao, R., Li, M., Zhang, D. & Xiao, H. Geochemistry and accumulation of the ultra-deep ordovician oils in the Shunbei oilfield, Tarim basin: coupling of reservoir secondary processes and filling events. *Mar. Pet. Geol.* **167**, 106959 (2024).

[60] Guo, X. et al. Effects of tectonic compression on petroleum accumulation in the Kelasu thrust belt of the Kuqa Sub-basin, Tarim Basin, NW China. *Org. Geochem.* **101**, 22–37 (2016).

[61] Lei, G. et al. Structural features and natural gas exploration in the Kelasu structural belt, Kuqa depression. *Oil Gas Geol.* **28**, 816–820 (2007).

[62] Liang, Q. Y., Xiong, Y. Q., Fang, C. C. & Li, Y. Quantitative analysis of diamondoids in crude oils using gas chromatography–triple quadrupole mass spectrometry. *Org. Geochem.* **43**, 83–91 (2012).

[63] Xuan, Y., Wang, W., Li, Y., Xiong, Y. & Jiang, W. M. Absolute quantitative analysis and thermal evolution of Trimantanes and tetramantanes in crude oil and source rock. *Geochimica* **53**, 643–654 (2024).

[64] Atwah, I. & Alsaif, M. Novel diamondoid detection technique using pMRM (GC-MS/MS): enabling source rock-oil-condensate correlations. *Org. Geochem.* **213**, 105125 (2026).

[65] Jiang, W. M., Li, Y., Fang, C. C., Yu, Z. Q. & Xiong, Y. Q. Diamondoids in petroleum: their potential as source and maturity indicators. *Org. Geochem.* **160**, 104298 (2021).

[66] Sweeney, J. J. & Burnham, A. K. Evaluation of a simple model of vitrinite reflectance based on chemical kinetics. *AAPG Bull.* **74**, 1559–1570 (1990).

[67] Gong, D. Y. et al. Geochemical characteristics and origins of the oils in Wushi Sag, Ratim basin. *Nat. Gas Geosci.* **25**, 62–69 (2014).

[68] Zhu, G. Y. et al. The geological feature and origin of Dina 2 large gas field in Kuqa Depression, Tarim basin. *Acta Petrolei Sinica*. **28**, 2479–2492 (2012).

[69] Li, J. et al. Source and exploration direction of tight oil and gas in the Dibei section of Northern Kuqa depression. *China Pet. Explor.* **24**, 485–497 (2019).

[70] Zhao, S. F., Chen, W. & Gao, Y. Analysis of fluid geochemical characteristics and accumulation process of Dibei condensate gas reservoir in Kuqa depression. *J. Xi’an Shiyou Univer (Nat. Sci. Ed)*. **37**, 1–10 (2022).

[71] Liu, R. H. et al. Geochemical characteristics and implication for gas and oil source correlation in the tugeerming area of the Kuqa Depression, Tarim basin. *Nat. Gas Geosci.* **30**, 574–581 (2019).

[72] Xiao, Z. Y., Huang, G., Lu, Y., Wu, Y. & Zhang, Q. Rearranged Hopanes in oils from the quele 1 Well, Tarim Basin, and the significance for oil correlation. *Pet. Explor. Dev.* **31**, 35–37 (2004).

[73] Liu, C. et al. Geochemical tracer of hydrocarbon migration path of Middle-Cenozoic in the South slope of the Kuqa foreland basin. *Acta Geol. Sinica*. **94**, 3488–3502 (2020).

[74] Jiang, W. M., Li, Y. & Xiong, Y. Q. The effect of organic matter type on formation and evolution of diamondoids. *Mar. Pet. Geol.* **89**, 714–720 (2018).

[75] Gordadze, G. N. Geochemistry of cage hydrocarbons. *Pet. Chem.* **48**, 241–253 (2008).

[76] Okui, A., Nishizuka, T. & Okamoto, S. Petroleum potential of Norwegian sedimentary basins revealed by detailed petroleum system analysis. *J. Jpn. Assoc. Pet. Technol.* **80**, 38–49 (2015).

[77] Fang, C. C., Xiong, Y. Q., Li, Y., Chen, Y. & Tang, Y. J. Generation and evolution of diamondoids in source rock. *Mar. Pet. Geol.* **67**, 197–203 (2015).

[78] Huang, W. Y. & Meinschein, W. G. Sterols as ecological indicators. *Geochem. Cosmochim. Acta*. **43**, 739–745 (1979).

[79] Fang, C. C., Xiong, Y. Q., Liang, Q. Y. & Li, Y. Variation in abundance and distribution of diamondoids during oil cracking. *Org. Geochem.* **47**, 1–8 (2012).

[80] van der Ploeg, R., Pureveen, J. B. M., van den Boorn, S. H. J. M. & van Bergen, P. F. Novel diamondoid-based maturity models using naturally occurring petroleum fluids. *AAPG Bull.* **107**, 1799–1810 (2023).

[81] Burnham, A. K. & Sweeney, J. J. A chemical kinetic model of vitrinite maturation and reflectance. *Geochim. Cosmochim. Acta*. **53**, 2649–2657 (1989).

[82] Jiang, W. M., Li, Y. & Xiong, Y. Q. Source and thermal maturity of crude oils in the Junggar basin in Northwest China determined from the concentration and distribution of diamondoids. *Org. Geochem.* **128**, 148–160 (2019).

[83] Berner, U. & Faber, E. Empirical carbon isotope/maturity relationships for gases from algal kerogens and terrigenous organic matter, based on dry, open-system pyrolysis. *Org. Geochem.* **24**, 947–955 (1996).

